# Dopants fixation of Ruthenium for boosting acidic oxygen evolution stability and activity

**DOI:** 10.1038/s41467-020-19212-y

**Published:** 2020-10-23

**Authors:** Shaoyun Hao, Min Liu, Junjie Pan, Xiangnan Liu, Xiaoli Tan, Nan Xu, Yi He, Lecheng Lei, Xingwang Zhang

**Affiliations:** 1 Key Laboratory of Biomass Chemical Engineering of Ministry of Education, College of Chemical and Biological Engineering, Zhejiang University, 310027 Hangzhou, Zhejiang Province China; 2 School of Physics and Electronics, Central South University, 410083 Changsha, Hunan China; 3 Institute of Zhejiang University-Quzhou, 78 Jiuhua Boulevard North, 324000 Quzhou, China

**Keywords:** Energy, Electrocatalysis, Nanoscale materials

## Abstract

Designing highly durable and active electrocatalysts applied in polymer electrolyte membrane (PEM) electrolyzer for the oxygen evolution reaction remains a grand challenge due to the high dissolution of catalysts in acidic electrolyte. Hindering formation of oxygen vacancies by tuning the electronic structure of catalysts to improve the durability and activity in acidic electrolyte was theoretically effective but rarely reported. Herein we demonstrated rationally tuning electronic structure of RuO_2_ with introducing W and Er, which significantly increased oxygen vacancy formation energy. The representative W_0.2_Er_0.1_Ru_0.7_O_2-δ_ required a super-low overpotential of 168 mV (10 mA cm^−^^2^) accompanied with a record stability of 500 h in acidic electrolyte. More remarkably, it could operate steadily for 120 h (100 mA cm^−^^2^) in PEM device. Density functional theory calculations revealed co-doping of W and Er tuned electronic structure of RuO_2_ by charge redistribution, which significantly prohibited formation of soluble Ru^x>4^ and lowered adsorption energies for oxygen intermediates.

## Abstract (web summary)

There is an increasing interest in understanding how defect chemistry can alter material reactivity. Here, authors tune the electronic structure of RuO_2_ by introducing W and Er dopants that boost acidic oxygen evolution performances by limiting oxygen vacancy formation during catalysis.

## Introduction

Electrochemical splitting water into H_2_ is considered as a promising technology for storing renewable and sustainable energy^1–7^. Acidic proton exchange membrane (PEM) water electrolyzers is highly desirable because the reaction rate in acidic electrolyte is three or more orders of magnitudes speedier than pH-neutral and alkaline electrolyzers owing to its higher voltage efficiency, more compact designing system, lower ohmic loss, and wider partial load range^8–11^. For decades, anode catalyst for PEM is mainly concentrated on IrO_2_ with the compromise between stability and activity in acidic electrolyte^12–17^. However, the low mass activity and the high cost extremely hindered its practical applications at large scale. Compared with IrO_2_, Ru-based materials usually exhibit the relatively higher activity owing to the suitable binding ability of oxygenated intermediate species (*OH, *O, and *OOH) with the surface active sites^18–22^, but with a poor stability. Recently, a series of Ru-based oxides, including oxide perovskites^23,24^, 3*d* metals doped RuO_2_^25^, heterostructured Ru@IrO_*x*_^3^ and Ru_1_–Pt_3_Cu^18^ with compressive strain, were explored to simultaneously enhance their activities and stabilities in acidic electrolyte by regulating the compositions and surface strain. Although the activities of Ru-based catalysts are attractive, the stability is still only dozens of hours in acidic electrolyte. It has been proven that the over-oxidation of Ru-based materials results in soluble Ru^*x*>4^ (e.g. RuO_4_) derivatives^14,26,27^, which seriously hinders the application of RuO_2_ in the commercial PEM devices.

The over-oxidation of Ru-based catalysts in acidic electrolyte is mainly caused by the oxidation of lattice oxygen, resulting in oxygen vacancies (V_O_) during OER^9,18,28^. The generation of V_O_ would expose Ru atoms on the surface^3,29–32^, which will be over-oxidized to soluble high valence Ru^*x*>4^ (e.g. RuO_4_) materials. The over-oxidation of Ru unavoidably leads to the collapse of the crystal structure, damaging the stability^28,29^. Therefore, it is reasonable to speculate that the over-oxidation of Ru-based catalysts could be thermodynamically hindered during OER if we can tune the electronic structure of RuO_2_ to make the formation energy of V_O_ much higher than the redox H_2_O/O_2_ energy. Foreign elements doping is the classical and effective strategy to enlarge the localized gap between O 2*p* band centers and Fermi level^28,33,34^. This would enhance the energy barrier for oxidation of lattice oxygen, prohibiting the formation of V_O_.

Herein, W and Er are co-doped into the lattice of RuO_2_ to modify its electronic structure, avoiding over-oxidation of Ru. By down-shifting O 2*p*-band centers, the energy (Δ*G*) for the V_O_ formation in W_*m*_Er_*n*_Ru_1−*m*−*n*_O_2−*δ*_ is significantly increased, preventing the generation of soluble high valence Ru^*x*>4^. As a result, the representative W_0.2_Er_0.1_Ru_0.7_O_2−*δ*_ offers a record low overpotential of 168 mV to achieve 10 mA cm^−^^2^ accompanied with a stability at least 500 h in 0.5 M H_2_SO_4_ electrolyte. It can also be applied as anode catalyst in acidic PEM with a high current of 100 mA cm^−^^2^ for over 120 h. Thus, tuning the electronic structure of RuO_2_ to prevent the over-oxidation and dissolution of Ru opens a feasible route to keep Ru^4+^ active sites for acidic OER. This work provides the insight to understand the stability of metal oxide catalysts in acidic electrolyte and a strategy to promote their activity and stability by fixing the catalysts.

## Results and discussion

### Mechanisms of W_0.2_Er_0.1_Ru_0.7_O_2−*δ*_ toward acidic OER

**Fig. 1:**
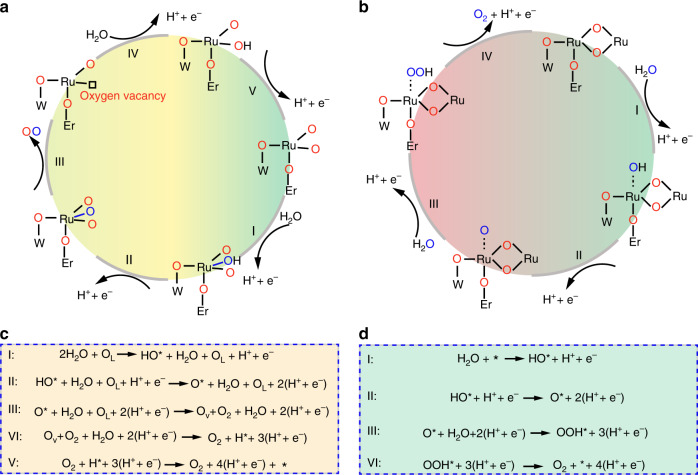
Schematic illustration of W_0.2_Er_0.1_Ru_0.7_O_2−*δ*_ toward acidic OER. **a** The illustration of lattice oxygen oxidation way of W_0.2_Er_0.1_Ru_0.7_O_2−*δ*_ toward acidic OER. **b** The illustration of adsorbate evolution way of W_0.2_Er_0.1_Ru_0.7_O_2−*δ*_ toward acidic OER. **c** the reaction paths of W_0.2_Er_0.1_Ru_0.7_O_2−*δ*_ for lattice oxygen oxidation. **d** the reaction paths of W_0.2_Er_0.1_Ru_0.7_O_2−*δ*_ for adsorbate evolution oxidation (* represents the surface-bound species on W_0.2_Er_0.1_Ru_0.7_O_2−*δ*_).

As for the metal dissolution in acidic electrolyte, it is mainly correlated with the adsorbate evolution and lattice oxygen oxidation mechanisms^9,18,35^. For lattice oxygen oxidation, there are four electrochemical steps as well as a non-electrochemical step for desorption of O_2_. In lattice oxygen oxidation route, the origin for releasing oxygen was occurred in step III (O* + H_2_O + O_L_ + 2(H^+^ + e^−^)→O_V_ + O_2_ + H_2_O + 2(H^+^ + e^−^), L represents lattice). With releasing oxygen from the lattice of electrocatalysts, the oxygen vacancies were created, in turn beneficially enhancing the OER activity (Fig. 1a, c)^28^. However, in acidic electrolyte, the existing oxygen vacancies would accelerate the over-oxidation of the exposed Ru, leading to the highly soluble Ru^*x*>4^ (e.g. RuO_4_) derivatives. Therefore, the structure of Ru-based materials on anodic side would be destroyed with releasing lattice oxygen from catalysts. As compared with lattice oxygen oxidation, there are only four electrochemical steps in the adsorbate evolution way toward OER (Fig. 1b)^9,36^. Simultaneously, in the adsorbate evolution route, O–O coupling from H_2_O in steps I and III was the origin for releasing O_2_ (step I: W_0.2_Er_0.1_Ru_0.7_O_2−*δ*_ + H_2_O → W_0.2_Er_0.1_Ru_0.7_O_2−*δ*_–OH* + H^+^ + e^−^, step III: W_0.2_Er_0.1_Ru_0.7_O_2−*δ*_–O* + H_2_O → W_0.2_Er_0.1_Ru_0.7_O_2−*δ*_–OOH* + H^+^ + e^−^) (Fig. 1d). The structures of catalysts would not be destroyed without releasing lattice oxygen. Therefore, effectively enhancing the energy for formation of oxygen vacancies and inhibiting the direct O–O coupling together from the lattice of W_0.2_Er_0.1_Ru_0.7_O_2−*δ*_ would be beneficial for maintaining the stability of Ru-based materials in acidic electrolyte (Fig. 1b, d).

### Density functional theory (DFT) calculations

**Fig. 2:**
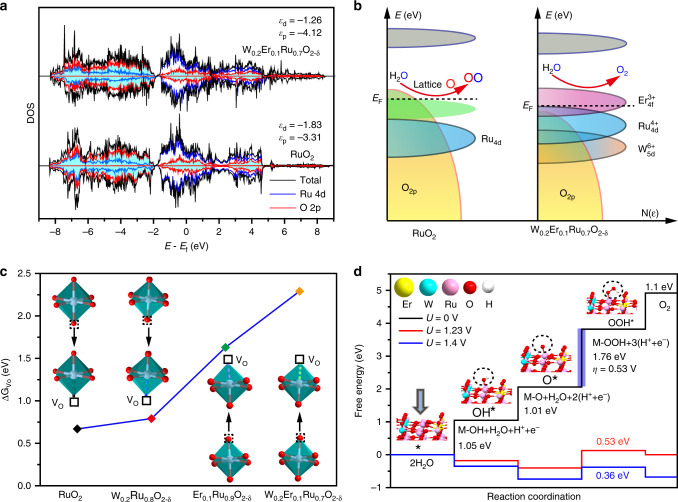
DFT for RuO_2_ and W_0.2_Er_0.1_Ru_0.7_O_2−*δ*_ toward acidic OER. **a** DOS plots of Ru 4*d* and O 2*p* states in RuO_2_ and W_0.2_Er_0.1_Ru_0.7_O_2−*δ*−1_. The dashed line means the Fermi level energy (*E*_F_). **b** Schematic diagrams of rigid band models for RuO_2_ and W_0.2_Er_0.1_Ru_0.7_O_2−*δ*−1_ toward acidic OER. **c** The calculated energy for formation of V_O_ in different positions of RuO_2_, W_0.2_Ru_0.8_O_2−*δ*−1_, Er_0.1_Ru_0.9_O_2−*δ*−1_, and W_0.2_Er_0.1_Ru_0.7_O_2−*δ*−1_. **d** The calculated energy barriers diagram for W_0.2_Er_0.1_Ru_0.7_O_2−*δ*−1_. The corresponding models of W_0.2_Er_0.1_Ru_0.7_O_2−*δ*−1_ to oxygen intermediates such as OH*, O* as well as OOH* (The number 1 or other numeral represents different established models for these structures).

In light of the reacted ways toward acidic OER, we carried out DFT studies aiming at rationally tuning the electronic of RuO_2_. To enhance the acidic OER stability, the energy for V_O_ formation should be thermodynamically suppressed. Density of states (DOS) from DFT was established to investigate how W and Er synergistically rationalize the Ru 4*d* and O 2*p* orbitals in RuO_2_ (Fig. 2a and Supplementary Fig. 1). The DOS spectra shows that O 2*p* band center (*ε*_p_) moves from −3.31 eV (RuO_2_) to −4.12 eV with W and Er introduction. And the gap between *ε*_p_ and Fermi level is obviously enlarged^28^. This result indicates the covalency of Ru–O bond is decreased by introduction of W and Er^37^. Therefore, in W_0.2_Er_0.1_Ru_0.7_O_2−*δ*_, the direct O–O coupling on O 2*p* states above the Fermi level will be not thermodynamically favored, which could be seen from Δ*G*_2_ for formation of OH* (Fig. 2b, right). This result will make Δ*G* for formation of V_O_ higher than that of RuO_2_ (Fig. 2b, left). Further, the DFT results also reveal Δ*G* for formation of V_O_ at the pyramid vertex of W_0.2_Er_0.1_Ru_0.7_O_2−*δ*_ shifts from lower energy band of 0.67 eV (RuO_2_) to higher energy band of 2.29 eV (Fig. 2c). These results indicate W and Er could effectively enhance the energy for formation of V_O_ in W_0.2_Er_0.1_Ru_0.7_O_2−*δ*_ toward acidic OER. Therefore, the dissolution of Ru in acidic electrolyte will be suppressed. Moreover, Bader charge analyses also prove the downshift of *p* band centers, resulting in the negative charge of the lattice O (Supplementary Fig. 2). Consequently, this electronic state could effectively enhance Δ*G* for the formation of V_O_ and suppress the lattice O binding with oxygen intermediates to release O_2_. Besides that, the corresponding model structures were established for oxidation of lattice oxygen on surface of (110) in W_0.2_Er_0.1_Ru_0.7_O_2−*δ*_ (Supplementary Fig. 3). However, the OH* adsorbed on W_0.2_Er_0.1_Ru_0.7_O_2−*δ*_ was not stable and transferred to Ru, W, or Er sites after optimization of the models (step II in Fig. 1c and Supplementary Fig. 3). Therefore, the lattice oxygen in W_0.2_Er_0.1_Ru_0.7_O_2−*δ*_ will not participate in acidic OER process. This result also confirmed that introducing W and Er into RuO_2_ could suppress the dissolution rate of Ru.

DFT calculations were then performed to rationalize the acidic OER performance (Fig. 2d and Supplementary Figs. 4–12). As also motived by the experimental results of synthesized samples, all the models were established based on (110) surface. Besides that, the solvation was not considered in our calculation because the DFT was intended to understand trends when doping the RuO_2_ surface with foreign metal atoms. All potential determining steps (PDS) for these designed samples were between OOH* and O*. The calculated Δ*G* for PDS followed the order of W_0.2_Er_0.1_Ru_0.7_O_2−*δ*−1_ (0.53 eV) < W_0.2_Ru_0.8_O_2−*δ*−1_ (0.6 eV) < Er_0.1_Ru_0.9_O_2−*δ*−1_ (0.72 eV) < RuO_2_ (0.79 eV) (Fig. 2d and Supplementary Figs. 4–6), revealing that simultaneously doping W and Er could reduce energy barriers for boosting activities. This phenomenon also agreed well with the upshift of Ru 4*d* band centers, which tuned the adsorption energy of oxygen intermediates on active sites. Moreover, the surface of the prepared and established models for these RuO_2_-based materials was not bare anymore at *U* > 1.23 V vs. RHE. And there should be neighboring intermediates around active sites, which influenced on the energetics of the elementary processes in the OER (Supplementary Figs. 9–11). According to the calculations, the energy barrier for PDS significantly decreased (Supplementary Figs. 10 and 11), compared with the traditional models without considering the neighboring intermediates (Supplementary Figs. 4–6). Therefore, these results also indicated that the neighboring intermediates around active sites could also contribute to enhancing the activity of W_0.2_Er_0.1_Ru_0.7_O_2−*δ*−1_ toward OER. Besides that, Δ*G*_2_ for formation of OH* has always acted as a descriptor for indicating the activity trends of the modeled materials^38^. The scaling relation of Δ*G*_2_ and Δ*G*_3_ for formation of OH* and OOH* should be also coupled: 2.6 eV ≤ Δ*G*_2_ + Δ*G*_3_ ≤ 3.6 eV^39^. As seen from the relationship of overpotential and Δ*G*_2_, the calculated Δ*G*_2_ for W_0.2_Er_0.1_Ru_0.7_O_2−*δ*−1_ (1.01 eV) was the apex of the volcano (Supplementary Fig. 12). Additionally, the calculated Δ*G*_2_ for W_0.2_Er_0.1_Ru_0.7_O_2−*δ*−1_ (1.01 eV) was also close to the theoretical Δ*G*_2_ (1.23 eV)^22^. These results revealed that W_0.2_Er_0.1_Ru_0.7_O_2−*δ*−1_ possessed the moderate Δ*G*_2_ among these structures toward acidic OER. According to above results, the co-doping of W and Er into RuO_2_ could effectively reduce generation of V_O_ and soluble Ru^*x*>4^, which sufficiently enhance the acidic OER stabilities and activities.

### Synthesis and characterization

**Fig. 3:**
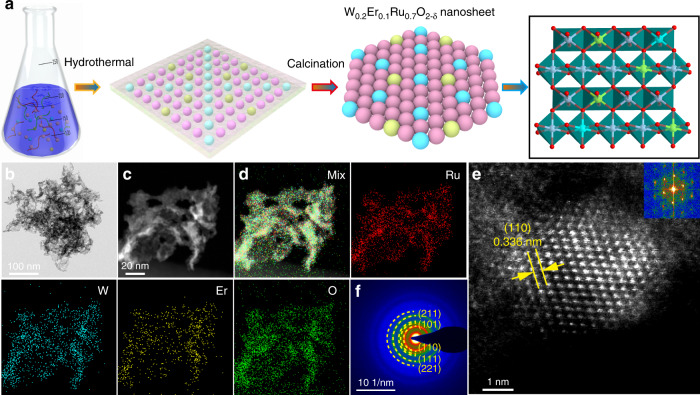
The designing strategy and TEM characterization for the prepared W_0.2_Er_0.1_Ru_0.7_O_2−*δ*_ nanosheets. **a** Schematic route for synthesis of W_0.2_Er_0.1_Ru_0.7_O_2−*δ*_ nanosheets. **b** TEM image, **c** HAADF-TEM image, **d** the corresponding elemental maps, **e** HR-TEM image (inset: Fourier transform analyses for W_0.2_Er_0.1_Ru_0.7_O_2−*δ*_), and **f** SAED image for the W_0.2_Er_0.1_Ru_0.7_O_2−*δ*_ nanosheets.

To experimentally probe the predictions, W_*m*_Er_*n*_Ru_1−*m*−*n*_O_2−*δ*_ nanosheets with various ratios were synthesized by a hydrothermal method, in which ethylenediaminetetraacetic acid (EDTA), ruthenium(III) acetate, WCl_6_, ErCl_3_, and citric acid were used as precursors (Fig. 3a). Supplementary Fig. 13 shows that these as-synthesized materials are of the rutile ruthenium oxides (RuO_2_, PDF#:40-1290) along with the dominant (110) peak. Transmission electron microscopy (TEM) exhibited that the prepared W_*m*_Er_*n*_Ru_1−*m*−*n*_O_2−*δ*_ samples possessed the ultrathin nanosheet morphology (Fig. 3b, c, and Supplementary Figs. 14–19). The corresponding elemental mapping confirmed that Er, W, and Ru were uniformly distributed in these nanosheets (Fig. 3d). High-resolution TEM (HR-TEM), fast Fourier transform (FFT), and selected area electron diffraction (SAED) revealed that W_0.2_Er_0.1_Ru_0.7_O_2−*δ*_ nanosheets possessed a face centered cubic (FCC) crystal face along (110) direction (Fig. 3e, f). The atomic ratios of Ru, W, and Er in these as-prepared samples were analyzed by inductively coupled plasma mass spectrometry (ICP-MS). The results revealed the atomic ratio of these elements was close to the expected values (Supplementary Tables 1–6).

### Oxidation state analysis

**Fig. 4:**
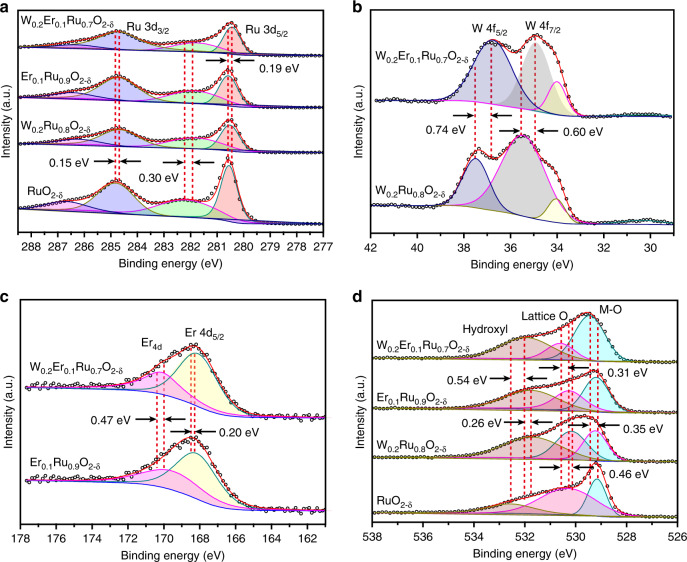
XPS characterization for W_0.2_Er_0.1_Ru_0.7_O_2−*δ*_, Er_0.1_Ru_0.9_O_2−*δ*_, W_0.2_Ru_0.8_O_2−*δ*_, and RuO_2−*δ*_ nanosheets. **a** Ru 3*d* spectra, **b** W 4*f* spectra, **c** Er 4*d* spectra, **d** O 1*s* spectra for the prepared W_0.2_Er_0.1_Ru_0.7_O_2−*δ*_, Er_0.1_Ru_0.9_O_2−*δ*_, W_0.2_Ru_0.8_O_2−*δ*_, and RuO_2−*δ*_ nanosheets. In order to precisely analyze the valance state of Ru, X-ray absorption near-edge spectroscopy (XANES) was applied to characterize RuO_2−*δ*_ and W_0.2_Er_0.1_Ru_0.7_O_2−*δ*_. Ru foil and C-RuO_2_ were employed as reference materials^3,18^. Compared with Ru K-edge position of Ru foil, the C-RuO_2_, RuO_2−*δ*_, and W_0.2_Er_0.1_Ru_0.7_O_2−*δ*_ all shifted to higher energy, resulting from the Ru–O bonds in these materials (Fig. 5a). Additionally, the Ru K-edge spectra in Fig. 5a showed the adsorption energy of the prepared RuO_2−*δ*_ and W_0.2_Er_0.1_Ru_0.7_O_2−*δ*_ were different from that for C-RuO_2_. This result mainly resulted from the fact that Ru valence state in RuO_2−*δ*_ and W_0.2_Er_0.1_Ru_0.7_O_2−*δ*_ were mainly dominated Ru^4+^ accompanied with Ru^*x*+^ (*x* < 4)^3^. Simultaneously, compared with adsorption energy of RuO_2−*δ*_, the adsorption energy for W_0.2_Er_0.1_Ru_0.7_O_2−*δ*_ shifted to lower energy region, indicating that Ru valence state in W_0.2_Er_0.1_Ru_0.7_O_2−*δ*_ was a little lower than that in RuO_2−*δ*_ due to introduction of W and Er. Additionally, the adsorption energy (*E*_0_) for RuO_2−*δ*_ (22,119.99 eV) was also a little higher than that for W_0.2_Er_0.1_Ru_0.7_O_2−*δ*_ (22,118.92 eV). These results were consistent with the valence state analysis in XPS. Furthermore, extended X-ray absorption fine structure (EXAFS) with Fourier transform as well as its counterpart (*k*^3^-weighted EXAFS) was applied to analyze the structure of RuO_2−*δ*_ and W_0.2_Er_0.1_Ru_0.7_O_2−*δ*_ (Fig. 5b). Compared with the bond of Ru–Ru in Ru foil (2.68 Å), W_0.2_Er_0.1_Ru_0.7_O_2−*δ*_ exhibited a slightly longer interatomic distance (2.71 Å), which could be related with the strained effect in HRTEM^3^ (Supplementary Table 7). Additionally, the Ru–Ru and Ru–O bonds in RuO_2−*δ*_ and W_0.2_Er_0.1_Ru_0.7_O_2−*δ*_ showed different interatomic distances is due to the existence of lower Ru^*x*<4^ valence state, compared with that in C-RuO_2_ (3.12 and 3.56 Å). Besides that, the different Ru–Ru, Ru–O bonds between RuO_2−*δ*_ and W_0.2_Er_0.1_Ru_0.7_O_2−*δ*_ should be related with introducing W and Er into RuO_2−*δ*_. Furthermore, wavelet transform (WT) for Ru K-edge EXAFS in Fig. 5c–f was applied to exhibit the length changes of Ru–Ru and Ru–O bonds in W_0.2_Er_0.1_Ru_0.7_O_2−*δ*_. The intensities at ≈6.5 Å^−1^ increased gradually, indicating the Ru^*x*<4^ had strong influence on W_0.2_Er_0.1_Ru_0.7_O_2−*δ*_, compared with that for C-RuO_2_. Besides that, compared with RuO_2−*δ*_, the intensities changed slightly at ≈13.5 Å^−1^ in W_0.2_Er_0.1_Ru_0.7_O_2−*δ*_ is due to the coordination of Ru–W/Er.

X-ray photoelectron spectra (XPS) was carried out on these prepared Ru-based nanosheet catalysts to analyze their compositions and chemical states (Supplementary Fig. 20). From Fig. 4a, the Ru 3*d* spectra for RuO_2−*δ*_, W_0.2_Ru_0.8_O_2−*δ*_, Er_0.1_Ru_0.9_O_2−*δ*_, and W_0.2_Er_0.1_Ru_0.7_O_2−*δ*_ could be deconvoluted into two kinds of doublets. The binding energies of lower energy couples situated at 280.6 (Ru 3*d*_5/2_) and 284.8 eV (Ru 3*d*_3/2_) were, respectively, attributed to Ru^*x*<4^ and Ru^4+^ ^3,40^. Simultaneously, the higher energy couples situated at 282.2 and 286.7 eV were, respectively, ascribed to the satellite peaks. The average area and intensity ratios for Ru 3*d*_3/2_ and Ru 3*d*_5/2_ were, respectively, higher (2:1.2) and lower (2:3.3) than the expected value of 2:3. This should be ascribed to the fact that the C 1*s* peak was coincided with Ru 3*d*^41^. The Ru 3*d*_3/2_ for W_0.2_Er_0.1_Ru_0.7_O_2−*δ*_ was negatively shifted (0.15 eV) compared with those of RuO_2−*δ*_, W_0.2_Ru_0.8_O_2−*δ*_, and Er_0.1_Ru_0.9_O_2−*δ*_, demonstrating that Ru in W_0.2_Er_0.1_Ru_0.7_O_2−*δ*_ was in a little lower valence state than Ru^4+^. For W 4*f*, the binding energies were deconvoluted into W 4*f*_5/2_ (37.4 eV) and W 4*f*_7/2_ (35.5 eV), indicating the element of W was in an valence state of W^6+^ (Fig. 4b)^42^. Similarly, the average area and intensity ratios for W 4*f*_5/2_ and W 4*f*_7/2_ doublet were, respectively, 15:17 and 9:10, which were close to the expected value of 3:4. Moreover, compared with W_0.2_Ru_0.8_O_2−*δ*_, the binding energies of W 4*f*_5/2_ and W 4*f*_7/2_ for W_0.2_Er_0.1_Ru_0.7_O_2−*δ*_ were, respectively, negatively shifted with 0.74 and 0.60 eV (Fig. 4b), confirming that W in W_0.2_Er_0.1_Ru_0.7_O_2−*δ*_ exhibited a lower valence state than W^6+^. From Fig. 3c, the binding energies for Er 4*d* could be fitted with two peaks, which should be, respectively, assigned to Er 4*d* (170.0 eV) and Er 4*d*_5/2_ (168.5 eV), revealing the Er element in these samples were in Er^3+^ valence state^43^. Interestingly, as compared with Er_0.1_Ru_0.9_O_2−*δ*_, the Er 4*d*_5/2_ and Er 4*d* for W_0.2_Er_0.1_Ru_0.7_O_2−*δ*_ were, respectively, positively shifted with 0.20 and 0.47 eV (Fig. 4c). This indicates that Er^3+^ in W_0.2_Er_0.1_Ru_0.7_O_2−*δ*_ was a little more positively charged. Besides that, O 1*s* peaks around at 532, 530.5, and 529.3 eV are, respectively, confirmed as hydroxyl groups, lattice oxygen, and M–O bonds. The lattice oxygen and M–O bonds in W_0.2_Er_0.1_Ru_0.7_O_2−*δ*_, W_0.2_Ru_0.8_O_2−*δ*_, and Er_0.1_Ru_0.9_O_2−*δ*_ nanosheets were slightly positive shifted compared with RuO_2−*δ*_, respectively (Fig. 4d), indicating O 1*s* was positively charged^44^. Through above analyses, it could be concluded that some electrons were transferred from Er to Ru and W through O in W_0.2_Er_0.1_Ru_0.7_O_2−*δ*_, confirming the electronic interactions among Ru, W, Er, and O^45^.

**Fig. 5:**
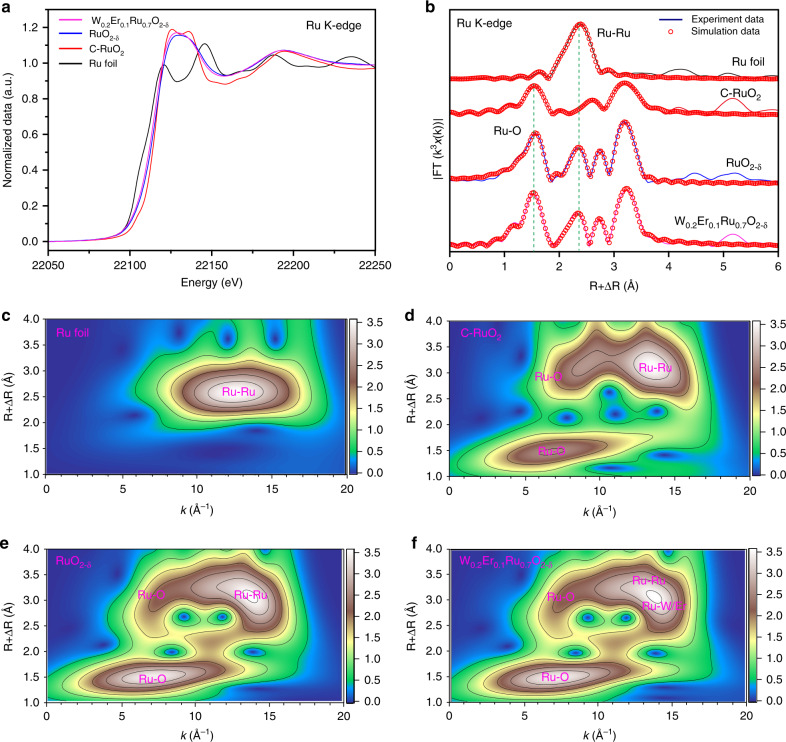
XANES characterization for W_0.2_Er_0.1_Ru_0.7_O_2−*δ*_ and RuO_2−*δ*_ nanosheets. **a** Ru K-edge spectra for Ru foil, C-RuO_2_, W_0.2_Er_0.1_Ru_0.7_O_2−*δ*_, and RuO_2−*δ*_. **b** FT-EXAFS spectra of Ru K-edge for Ru foil, C-RuO_2_, W_0.2_Er_0.1_Ru_0.7_O_2−*δ*_, and RuO_2−*δ*_. **c-f** WT-EXAFS of Ru foil, C-RuO_2_, RuO_2−*δ*_, and W_0.2_Er_0.1_Ru_0.7_O_2−*δ*_, respectively.

### Electrochemical OER in acid electrolyte

**Fig. 6:**
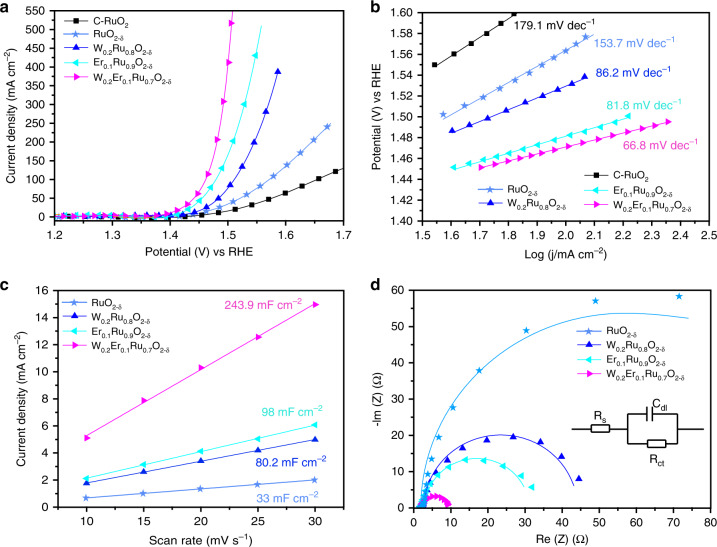
Acidic OER activities for these samples tested in 0.5 M H_2_SO_4_ electrolyte. **a** Polarization curves, **b** corresponding Tafel slopes calculated from **a**. **c** *C*_dl_ plots inferred from CV curves. **d** EIS plots for W_0.2_Er_0.1_Ru_0.7_O_2−*δ*_, Er_0.1_Ru_0.9_O_2−*δ*_, W_0.2_Ru_0.8_O_2−*δ*_, and RuO_2−*δ*_ nanosheets.

To establish correlation between the electrocatalytic performances and the specific electronic structure, the OER activities of the prepared W_*m*_Er_*n*_Ru_1−*m*−*n*_O_2−*δ*_ nanosheets and C-RuO_2_ were investigated in 0.5 M H_2_SO_4_ (Supplementary Figs. 21 and 22). The linear sweep voltammetry (LSV, scan rate: 5 mV s^−^^1^) was corrected with *iR* and normalized with geometrical area, respectively. It was seen that the performances of prepared electrocatalysts followed an order of W_0.2_Er_0.1_Ru_0.7_O_2−*δ*_ > Er_0.1_Ru_0.9_O_2−*δ*_ > W_0.2_Ru_0.8_O_2−*δ*_ nanosheets > C-RuO_2_ NPs as shown in Fig. 6a. Simultaneously, the representative W_0.2_Er_0.1_Ru_0.7_O_2−*δ*_ nanosheet exhibited a record performance toward OER with an overpotential (*ɳ*) as low as 168 mV at 10 mA cm^−^^2^ (Fig. 6a), remarkably preceding other prepared Ru-based nanosheets. Simultaneously, W_0.2_Er_0.1_Ru_0.7_O_2−*δ*_ also exhibited the fastest reaction rate among these prepared samples with a small Tafel slope of 66.8 mV dec^−^^1^ (Fig. 6b). The current density of W_0.2_Er_0.1_Ru_0.7_O_2−*δ*_ nanosheets could achieve 500 mA cm^−^^2^ at *ɳ* = 275 mV, which was 28.6-folds higher than that of C-RuO_2_. It should be noted that the performance for the representative W_0.2_Er_0.1_Ru_0.7_O_2−*δ*_ was also superior to those of the catalysts reported recently (Supplementary Table 9).

To reveal the origin of the performances for prepared samples, electrochemical double-layer capacitance (*C*_dl_) was performed to measure the surface area and roughness factor (*R*_f_) (Fig. 6c and Supplementary Figs. 23–27). Moreover, CV curves for these materials supported on glass carbon electrode were also measured to confirm *C*_dl_ (Supplementary Figs. 26 and 27). As listed in Supplementary Table 8, the calculated *C*_dl_, *R*_f_, and surface area exhibited a similar trend as activity. W_0.2_Ru_0.8_O_2−*δ*_, Er_0.1_Ru_0.9_O_2−*δ*_, and W_0.2_Er_0.1_Ru_0.7_O_2−*δ*_ nanosheet structures revealed successively increased roughness compared with RuO_2−*δ*_ nanosheets. The representative W_0.2_Er_0.1_Ru_0.7_O_2−*δ*_ (1231.8 m^2^ g^−^^1^) exhibited much larger surface area than Er_0.1_Ru_0.9_O_2−*δ*_ (494.9 m^2^ g^−^^1^), W_0.2_Ru_0.8_O_2−*δ*_ (326.3 m^2^ g^−^^1^), and RuO_2−*δ*_ (166.7 m^2^ g^−^^1^), probably originating from more active sites exposure with co-doping of W and Er into RuO_2_ nanosheets (Supplementary Fig. 21d). The calculated specific activity followed an order of W_0.2_Er_0.1_Ru_0.7_O_2−*δ*_ (1.23 mA cm^−2^_ox_) > W_0.2_Ru_0.8_O_2−*δ*_ (1.16 mA cm^−^^2^_ox_) > Er_0.1_Ru_0.9_O_2−*δ*_ (1.15 mA cm^−^^2^_ox_) > RuO_2__−*δ*_ (0.98 mA cm^−^^2^_ox_) > C-RuO_2_ (0.50 mA cm^−^^2^_ox_). Simultaneously, W_0.2_Er_0.1_Ru_0.7_O_2−*δ*_ also possessed the highest mass activity of 1518.6 A g^−^^1^_ox_, which was 2.7, 5.8, and 28.5-folds higher than Er_0.1_Ru_0.9_O_2−*δ*_, W_0.2_Ru_0.8_O_2−*δ*_, and C-RuO_2_, respectively (Supplementary Fig. 21d). The highest specific and mass activities for W_0.2_Er_0.1_Ru_0.7_O_2−*δ*_ among these samples indicated the highest intrinsic activity of W_0.2_Er_0.1_Ru_0.7_O_2−*δ*_.

Nyquist plots in Fig. 6d were applied to fit the equivalent circuit diagram for the electrochemical impedance spectroscopy, revealing the reaction impendence between the electrode and solution. Figure 6d shows that the charge-transfer resistance (*R*_ct_) for W_0.2_Er_0.1_Ru_0.7_O_2-*δ*_ was 3.3 Ω, which was much smaller than those of Er_0.1_Ru_0.9_O_2−*δ*_ (13.8 Ω), W_0.2_Ru_0.8_O_2−*δ*_ (20.2 Ω), and RuO_2−*δ*_ (50.8 Ω). These results further confirmed the co-doping of W and Er into RuO_2−*δ*_ nanosheets enhanced the intrinsic OER property of the samples. Additionally, Faraday efficiency for OER on W_0.2_Er_0.1_Ru_0.7_O_2−*δ*_ could reach 99.5%, indicating the current mainly came from OER as shown in Supplementary Fig. 28.

### Stability for acidic OER and PEM

**Fig. 7:**
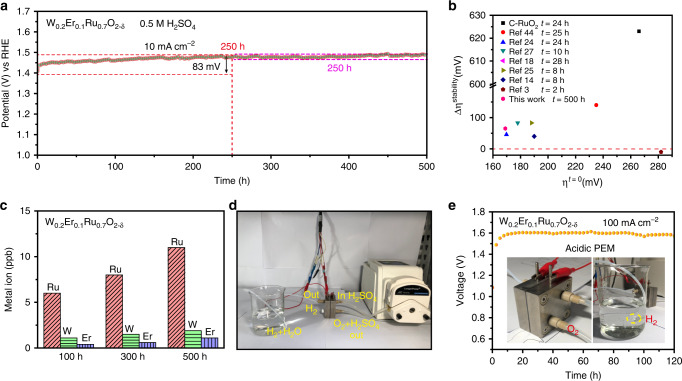
The long-durable stability investigation for OER and PEM in acidic electrolyte. **a** The current-time (500 h) stability of W_0.2_Er_0.1_Ru_0.7_O_2−*δ*_ nanosheets in 0.5 M H_2_SO_4_. **b** The comparison of stabilities in acidic electrolyte for various electrocatalysts, *x*-axis stands for the initial *ɳ* for electrocatalysts reaching 10 mA cm^−^^2^, *y-*axis represents the final *ɳ* of various electrocatalysts after stability measurement. **c** ICP analysis for W_0.2_Er_0.1_Ru_0.7_O_2−*δ*_ after 500 h operation in acidic electrolyte. **d** Photograph of the employed PEM reaction device. **e** The current–time (120 h) stability of W_0.2_Er_0.1_Ru_0.7_O_2−*δ*_ as anodic side in acidic PEM (inset: PEM reaction device, detection of H_2_).

The stability in acidic electrolyte is a critical factor to evaluate the performances of OER electrocatalysts due to highly oxidative operating conditions and corrosive electrolytes. The stability of W_0.2_Er_0.1_Ru_0.7_O_2−*δ*_ could be assessed by ∆*ɳ* = *ɳ*_final_–*ɳ*_initial_, the gap between the final and initial overpotential (*ɳ*) can be calculated from a chronopotentiometric test of 10 mA cm^−^^2^. From Fig. 7a, the chronopotentometric line exhibited a highly durable stability for 250 h with a small increase of ∆*ɳ* = 83 mV, indicating the excellent stability of W_0.2_Er_0.1_Ru_0.7_O_2−*δ*_ (Fig. 7b and Supplementary Table 9). Remarkably, from 250 to 500 h during the stability test, the operation ran stably with a tiny increase of ∆*ɳ* = 5 mV, confirming the durable properties of W_0.2_Er_0.1_Ru_0.7_O_2−*δ*_ in acidic electrolyte. Besides that, the stability of W_0.2_Er_0.1_Ru_0.7_O_2−*δ*_ loaded on glass carbon electrode also revealed the durable stability in acidic electrolyte (Supplementary Fig. 29). As a contrast, the C-RuO_2_ could only run 24 h with the large loss of ∆*ɳ* = 615 mV (Supplementary Fig. 30). To confirm the fixation of Ru in W_0.2_Er_0.1_Ru_0.7_O_2−*δ*_, ICP-MS was performed to analyze the concentration of the dissolved W, Er, and Ru in the solution during the 500 h stability test at an interval of 100 h (Fig. 7c). The results of ICP-MS revealed that the Ru cations in the solution were <12 ppb after 500 h (Supplementary Tables 10–18). More significantly, W_0.2_Er_0.1_Ru_0.7_O_2−*δ*_ as anodic electrocatalyst could also operate steadily for 120 h at high current of 100 mA cm^−^^2^ in acidic PEM (Fig. 7d, e).

### Charge redistribution of W_0.2_Er_0.1_Ru_0.7_O_2−*δ*_ for the long-durable acidic OER stability

**Fig. 8:**
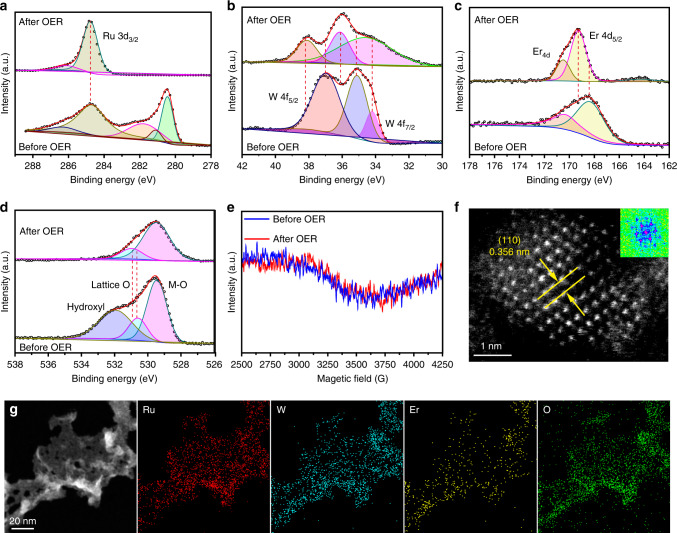
Chemical recognition and characterization for W_0.2_Er_0.1_Ru_0.7_O_2−*δ*_ after 500 h stability. **a** Ru 3*d* spectra, **b** W 4*f* spectra, **c** Er 4*d* spectra, **d** O 1*s* spectra for W_0.2_Er_0.1_Ru_0.7_O_2−*δ*_ before and after 500 h stability. **e** ESR spectra for W_0.2_Er_0.1_Ru_0.7_O_2−*δ*_ before and after 500 h stability. **f** HR-TEM image, **g** Elemental mapping for W_0.2_Er_0.1_Ru_0.7_O_2−*δ*_ after acidic OER stability.

To prove the highly improved dissolution and oxidation resistance of W_0.2_Er_0.1_Ru_0.7_O_2−*δ*_ toward OER in acidic electrolyte, the chemical states for Ru, W, Er, and O in W_0.2_Er_0.1_Ru_0.7_O_2−*δ*_ after the acidic OER measurement were further investigated and compared with these elements before OER, respectively (Fig. 8). The states of W_0.2_Er_0.1_Ru_0.7_O_2−*δ*_ after the 500 h stability test were analyzed by XPS. For Ru 3*d*, the peaks at 284.8 eV for Ru 3*d*_3/2_ was not obviously positively or negatively shifted as compared with that of the sample before OER test, suggesting that the valence of active sites Ru remained Ru^4+^ during OER test (Fig. 8a). Simultaneously, the peak at 280.6 eV (Ru 3*d*_5/2_) disappeared, indicating that the Ru^*x*<4^ were all oxidized to Ru^4+^.

Besides that, Ru valence state in W_0.2_Er_0.1_Ru_0.7_O_2−*δ*_ after OER was also characterized with both XANES and EXAFS (Supplementary Fig. 31). W_0.2_Er_0.1_Ru_0.7_O_2−*δ*_ showed similar adsorption energy with that for C-RuO_2_, indicating Ru stayed Ru^4+^. Simultaneously, compared with adsorption energy of W_0.2_Er_0.1_Ru_0.7_O_2−*δ*_ before OER, adsorption energy of W_0.2_Er_0.1_Ru_0.7_O_2−*δ*_ after OER shifted to higher energy, implying Ru^*x*<4^ were all oxidized to Ru^4+^. This result also implied that there was no RuO_4_ generation during acidic OER. Moreover, EXAFS spectra exhibited that the Ru–Ru bond (2.71 Å) in W_0.2_Er_0.1_Ru_0.7_O_2−*δ*_ disappeared after OER, indicating that the Ru^*x*<4^ were oxidized to Ru^4+^ (Supplementary Table 19). Simultaneously, the Ru–Ru, Ru–O, Ru–W/Er bonds in W_0.2_Er_0.1_Ru_0.7_O_2−*δ*_ after OER was very close to the W_0.2_Er_0.1_Ru_0.7_O_2−*δ*_, indicating the crystal structure was kept almost unchangeable, except for the lower Ru^*x*<4^.

The binding energies for W and Er in W_0.2_Er_0.1_Ru_0.7_O_2−*δ*_ after OER were positively shifted compared with those before OER, respectively. This suggested that W and Er in W_0.2_Er_0.1_Ru_0.7_O_2−*δ*_ after OER were positively charged (Fig. 8b, c). Compared with O 1*s* in W_0.2_Er_0.1_Ru_0.7_O_2−*δ*_ before OER, the lattice O and M–O groups in W_0.2_Er_0.1_Ru_0.7_O_2−*δ*_ after OER were slightly positively shifted, indicating O 1*s* were slightly positively charged after OER (Fig. 8d). Simultaneously, the ratio between the area of the lattice oxygen and M–O groups of W_0.2_Er_0.1_Ru_0.7_O_2−*δ*_ after OER stability became smaller, compared with that for W_0.2_Er_0.1_Ru_0.7_O_2−*δ*_ before OER. Additionally, the hydroxyl groups disappeared in W_0.2_Er_0.1_Ru_0.7_O_2−*δ*_ after OER, suggesting the hydroxyl groups were oxidized during OER. The surface rearrangement in W_0.2_Er_0.1_Ru_0.7_O_2−*δ*_ during acidic OER should be responsible for the shift in these electron states (Fig. 8a–d). Simultaneously, after the surface rearrangement, partial electrons from W and Er were transferred to Ru atoms, achieving a charge redistribution among Ru, W, Er, and O. The similar redistribution was also observed in IrO_*x*_/SrIrO_3_ for acidic OER^46^. Remarkably, after electron charge redistribution, Ru 4*d* still keeps Ru^4+^ without the generation of soluble Ru^*x*+^ (x > 4) derivatives. Therefore, charge redistribution among W, Er, Ru, and O is one reason for achieving the long-durable stability of W_0.2_Er_0.1_Ru_0.7_O_2−*δ*_ in acidic OER.

### Characterization of W_0.2_Er_0.1_Ru_0.7_O_2−*δ*_ after acidic OER

To prove there was no generation of oxygen vacancies on W_0.2_Er_0.1_Ru_0.7_O_2−*δ*_ during acidic OER, electron spin resonance (ESR) was further applied to characterize W_0.2_Er_0.1_Ru_0.7_O_2−*δ*_ after the stability test (Fig. 8e). ESR spectra revealed that there were no obvious peaks around 3275 G (oxygen vacancies) in W_0.2_Er_0.1_Ru_0.7_O_2−*δ*_, confirming that there were no generation of oxygen vacancies during acidic OER, directly proving co-doping of W and Er could enhance the energy formation of oxygen vacancies (Supplementary Fig. 2). Simultaneously, it also proved that the O–O coupling was from H_2_O, rather than from lattice oxygen (Fig. 1). Consequently, there was no generation of high-valance RuO_4_, avoiding collapsing the crystal structure of W_0.2_Er_0.1_Ru_0.7_O_2−*δ*_. Additionally, the morphology of the W_0.2_Er_0.1_Ru_0.7_O_2−*δ*_ remained intact after 500 h operation under acidic electrolyte (Fig. 8f, g). The fractional lattice spacing of W_0.2_Er_0.1_Ru_0.7_O_2−*δ*_ also kept at ~0.356 nm, which may be due to the surface rearrangement during acidic OER (Fig. 8f and Supplementary Fig. 32). The elemental mappings of W_0.2_Er_0.1_Ru_0.7_O_2−*δ*_ revealed that W, Er, Ru, and O were still distributed uniformly in the sample (Fig. 8g), suggesting the unchanged composition. Moreover, XRD pattern of W_0.2_Er_0.1_Ru_0.7_O_2−*δ*_ after OER did not change obviously, implying that the structure kept unchangeable (Supplementary Fig. 33).

In summary, we applied doping engineering with W and Er for simultaneously boosting the OER activities and stabilities of RuO_2_ in acidic electrolyte by tuning electronic structure of RuO_2_. The generation of oxygen vacancies during OER was thermodynamically difficult due to the obviously downshift O 2*p* band centers, which improved the dissolution and oxidation resistance of Ru. Remarkably, the representative W_0.2_Er_0.1_Ru_0.7_O_2−*δ*_ OER catalyst only required a 168 mV overpotential to reach 10 mA cm^−^^2^ accompanied with long-durable stability of 500 h in acidic electrolyte, overwhelmingly outperforming other Ru-based electrocatalysts reported so far. Particularly, the obtained sample could also operate stably for 120 h at a high current of 100 mA cm^–2^ in acidic PEM, further pushing its applications in industrial field. This study not only offers deep insights into tuning electronic properties of catalysts to enhance stabilities, but also opens a new-record horizon for designing electrocatalysts with super activity applying in acidic PEM.

## Methods

*Materials*: Ruthenium(III) acetate (C_14_H_21_O_14_Ru_3_), Tungsten(VI) chloride (WCl_6_), and Erbium(III) chloride (ErCl_3_) were all directly obtained from Aladdin Reagent (Shanghai) Co., Ltd. HCl and H_2_SO_4_ was purchased from Sinopharm Co., Ltd. EDTA and citric acid were provided with Shanghai Macklin Biochemical Co., Ltd. Carbon paper (CP) was supplied with Shanghai Hesen Electric Co., Ltd. Ultra-pure (18.2 MΩ cm^−1^) water was applied to deal with the prepared electrodes and electrolytes.

### Preparation of RuO_2−*δ*_, W_0.1_Ru_0.9_O_2−*δ*_, W_0.2_Ru_0.8_O_2−*δ*_, W_0.3_Ru_0.7_O_2−*δ*_, Er_0.1_Ru_0.9_O_2−*δ*_, W_0.2_Er_0.1_Ru_0.7_O_2−δ_, and W_0.2_Er_0.2_Ru_0.6_O_2−*δ*_ nanosheets

All samples were prepared by the hydrothermal method. Firstly, C_14_H_21_O_14_Ru_3_ (0.07 mol, 5.02 g), WCl_6_ (0.002 mol, 0.79 g), and ErCl_3_ (0.001 mol, 0.27 g) were added into ultra-pure water with vigorous stirring. Then, EDTA (0.01 mol, 2.92 g) and citric acid (0.01 mol, 1.92 g) was added into ultra-pure water with regulating pH to 9 by NH_3_·H_2_O. Finally, the solution containing EDTA and critic acid was added drop-wise into the metal salt solution for reaction for 12 h at 353 K. The obtained precursors were tiled in a treated porcelain boat and heated to 573 K for 4 h and 673 K for 2 h with a heating rate of 5 K/min in muffle furnace, respectively. The porous W_0.2_Er_0.1_Ru_0.7_O_2−*δ*_ nanosheets were obtained after the muffle furnace cooling to 303 K. The methods for preparation of the RuO_2−*δ*_, W_0.1_Ru_0.9_O_2−*δ*_, W_0.2_Ru_0.8_O_2−*δ*_, W_0.3_Ru_0.7_O_2−*δ*_, Er_0.1_Ru_0.9_O_2−*δ*_, and W_0.2_Er_0.2_Ru_0.6_O_2−*δ*_ nanosheets were the same as that of W_0.2_Er_0.1_Ru_0.7_O_2−*δ*_ nanosheets.

### Characterization

The XRD patterns for prepared electrocatalysts were all obtained on a X-pert Powder (PANalytical B.V., Netherlands) with PIXcel 1*d* detector and Cu-K*α* (*λ* = 1.54178 Å) radiation. Additionally, Tecnai G2 F20 S-TWIN (FEI, America) was used to obtain the TEM, HR-STEM, and elemental mapping at a voltage of 200 kV. For XPS analysis, X-ray photoelectron spectrometer (Thermo Scientific Escalab Xi+, England) was used to characterize these representative electrocatalysts. For ESR, JEOL FA200 was applied to characterize these samples. ICP-MS (Agilent ICP-OES730) was employed to show the ratios of these elements in W_0.2_Er_0.1_Ru_0.7_O_2−*δ*_. BL01C1 Beamline in NSRRC was employed to collect X-ray absorption spectra. Simultaneously, a double-crystal monochromator (Si, 111) was monochromatized for the radiation. Athena software was employed to analyze the XANES, EXAFS data.

### Electrochemical measurements

These prepared electrodes were characterized by Bio-Logic VSP potentiostat. The mass-loading for prepared electrocatalysts on CP was 0.33 mg cm^–2^. The prepared C-RuO_2_/CP, RuO_2−*δ*_/CP, W_0.1_Ru_0.9_O_2−*δ*_/CP, W_0.2_Ru_0.8_O_2−*δ*_/CP, W_0.3_Ru_0.7_O_2−*δ*_/CP, Er_0.1_Ru_0.9_O_2−*δ*_/CP, and W_0.2_Er_0.1_Ru_0.7_O_2−*δ*_/CP, W_0.2_Er_0.2_Ru_0.6_O_2−*δ*_/CP (1 × 0.5 cm^2^) were, respectively, applied as working electrodes. Meanwhile, the platinum column electrode and Hg/Hg_2_SO_4_ (0.645 V) were, respectively, employed as counter and reference electrodes. Besides that, LSV with *i*R correction was applied to characterize activities of these samples in electrolytic cell (40 mL, 0.5 M H_2_SO_4_) with purging O_2_ about 30 min. The EIS was tested in a range of 0.01–100 kHz at onset potential and open circuit voltage. The stabilities of C-RuO_2_ and W_0.2_Er_0.1_Ru_0.7_O_2−*δ*_ were carried out at 10 mA cm^–2^ toward OER. Additionally, the catalysts loaded on new glass carbon electrodes were employed for the stability test at 10 mA cm^–2^ or 1.4 V vs. RHE.

The measurement of PEM was performed on the self-made cell, which was composed of two SS316 steel plates. In PEM, the anodic W_0.2_Er_0.1_Ru_0.7_O_2−*δ*_ catalyst and cathodic Pt/C (20%, commercial) were, respectively, sprayed on Ti foam and CP (2 × 2 cm^2^), respectively. Additionally, the Ti foam and CP-loaded electrocatalysts were fixed with N117 membrane together using hot pressing at 363 K for 30 min. 0.5 M H_2_SO_4_ (3 mL min^–1^) was applied as electrolyte during the test.

Double layered capacitances (*C*_dl_) for these samples were assessed by CV with a san rate from 10 to 30 mV s^–1^ at a window of 1.01–1.11 V vs. RHE. The *C*_dl_ can be calculated from the scan rates with current densities (*j*) obtaining from CV curves. Simultaneously, specific capacitance of 40 μF cm^−2^ was applied to assess the ECSA. Additionally, the catalysts loaded on new glass carbon electrodes were also employed for the CV test.

### Faradaic efficiency (FE)

FE for W_0.2_Er_0.1_Ru_0.7_O_2−*δ*_ toward acidic OER (0.5 M H_2_SO_4_) was measured with the three-electrode configuration as well as detected by a GC-9790II gas chromatography. The column is 5 A molecular sieve (length: 2 m, diameter: 3 mm). The detector is thermal conductivity detector (TCD). The electrolyte under stirring was firstly degassed by Ar gas for half an hour. Then, the gaseous sample was taken out with a gas tight syringe every 20 min at 10 mA. Additionally, the samples were analyzed via a GC calibrated O_2_. Each injection into GC was repeated to eliminate error.

FE could be calculated through Eq.(1):

1 $${\mathrm{FE}}\left( {{\mathrm{O}}_2{\mathrm{\% }}} \right) = \frac{{V_{{\mathrm{O}}2} \ast 4 \ast F}}{{V_{\mathrm{m}} \ast i \ast t \ast 100\% }},$$

where *V*_O2_ represents the volume of O_2_, *F* represents Faraday constant (96,485.33289 C/mol), *i* stands for the applying current, *t* is the total time for OER, and *V*_m_ represents the molar volume of the generated O_2_.

### Theoretical calculations

Spin-polarized DFT calculations were conducted on projector-augmented wave (PAW)^47^ in the Vienna Ab initio Simulation Package (VASP)^48^. The generalized gradient approximation (GGA) of Perdew–Burke–Ernzerhof (PBE)^49^ exchange-functional functional was applied. The cut-off energy for plane-wave basis was set as 450 eV. A 20 Å vacuum slab in a direction perpendicular to the surface of catalyst was adopted to avoid periodic interactions. The Brillouin zone integration was performed with 3 × 3 × 1 Monkhorst–Pack *k*-point sampling for geometry relaxation^50^. For the calculations of DOS, the *k*-point mesh was increased to 6 × 6 × 1. The convergence threshold for force and energy during optimization were set as 0.03 eV/Å, 10^–4^ eV, respectively. A *p*(4 × 4) unit cell of RuO_2_ (110) surface with three layers in their slab was comprised. In the maintext and supplementary information, the influence of neighboring intermediates on the energetics was not considered when free-binding energies were calculated without special instructions. Additonally, the influence of aqueous solvent in calculation were not considered. The first two layers as well as the adsorbate intermediates were relaxed with the fixation of other atoms for geometry optimization. For Er and W doping, three different initial structures are considered, +3 and +6 valences of Er and W are taken into account, respectively. It is worth mentioning that the model of Er-doped exhibited a relatively lower energy as compared with Er and W co-doped, because of the larger distortion of the adjacent O at the Er defect position, another more reasonable structural model is selected. On this basis, we simulated the adsorption behavior of *O, *OH, and *OOH intermediates for each catalyst, and each model was optimized to convergence. Δ*G* for each OER step was calculated through the model of computational hydrogen electrode^51^ along with the equation as following:

2 $$\Delta G = \Delta E_{{\rm{{ZPE}}}} + \Delta E--T \times \Delta S.$$

The formula for calculation of V_O_:

3 $$\Delta {G}\left( {{V}_{\mathrm{O}}} \right) = {G}\left( {{V}_{\mathrm{O}}} \right) + \left( {{G}_{{\mathrm{H2O}}}-{G}_{{\mathrm{H2}}}} \right)-{G}\left( { \ast {\mathrm{O}}} \right),$$

where Δ*E* refers to DFT energy difference; Δ*S* and Δ*E*_ZPE_ refer to corrections with entropy through vibrational analysis and zero point energy at 300 K, respectively; *G*(*V*_O_) refers to the energy of the structure after leaving a vacancy; *G*(*O) refers to the energy that adsorbs the structure of *O intermediate; *G*_H2O_ and *G*_H2_ are the energy for water molecules and hydrogen molecules, respectively.

## Supplementary information

Supplementary information

## Data Availability

All of the authors claim that the presented data in this work will be available for the contacts from corresponding author with reasonable request.
