# Supplementary material for: Dopants fixation of Ruthenium for boosting acidic oxygen evolution stability and activity
Source: Nat Commun. 2020 Oct 23;11:5368. doi: 10.1038/s41467-020-19212-y (PMC7584605; doi:10.1038/s41467-020-19212-y)
Supplement: Supplementary file 1 — Supplementary information [file 41467_2020_19212_MOESM1_ESM.pdf]

Supporting Information for:

Dopants fixation of Ruthenium for boosting acidic oxygen evolution stability and activity

Hao etal.

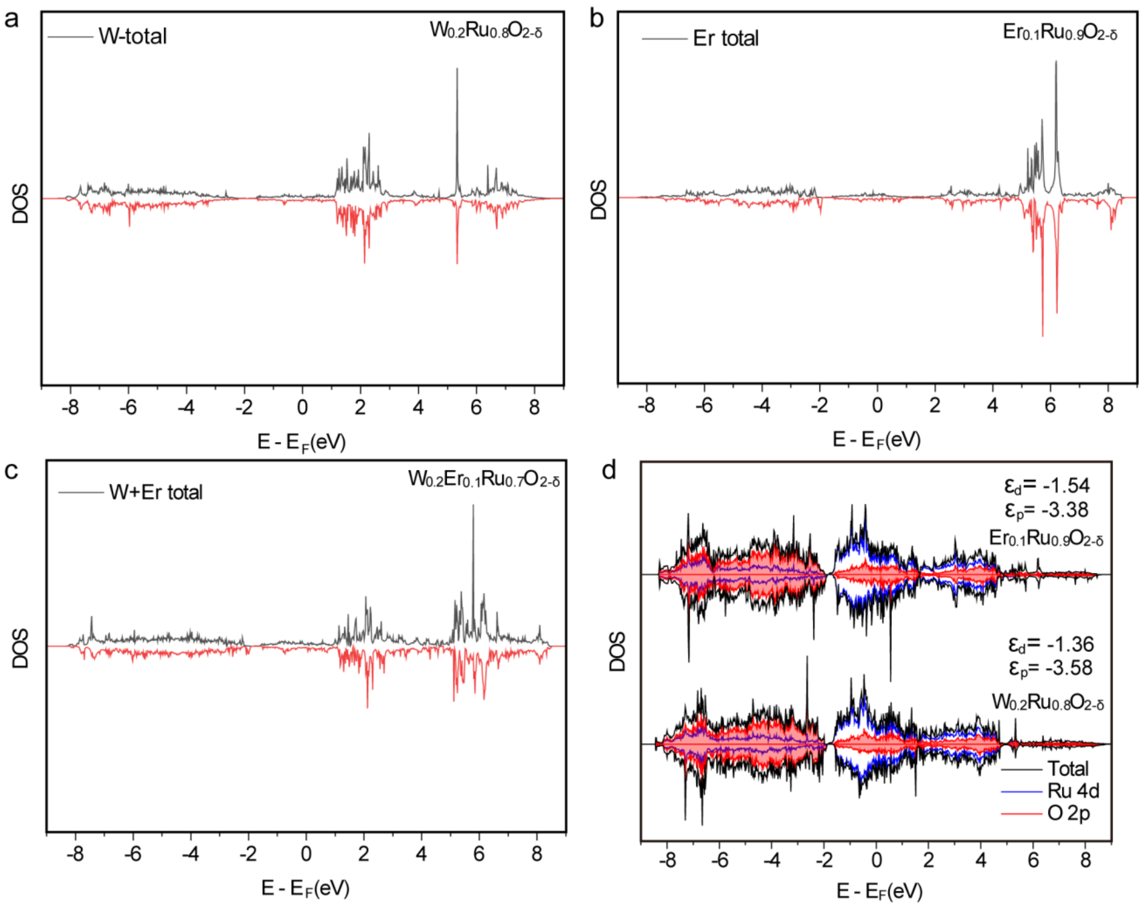

Supplementary Figure 1. **DOS plots for the established models**. DOS plots for (a) W in $W_{0.2}Ru_{0.8}O_{2-\delta}$, (b) Er in $Er_{0.1}Ru_{0.9}O_{2-\delta}$, and (c) W and Er in $W_{0.2}Er_{0.1}Ru_{0.7}O_{2-\delta}$. (d) DOS plots of Ru 4d and O 2p states in $W_{0.2}Ru_{0.8}O_{2-\delta}$ and $Er_{0.1}Ru_{0.9}O_{2-\delta}$.

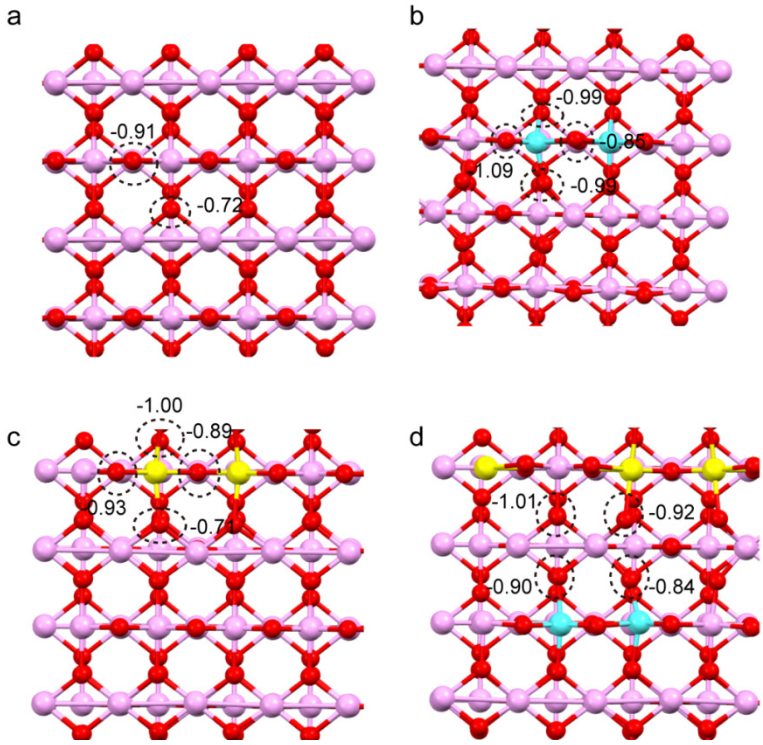

Supplementary Figure 2. **The Bader charge around Ru sites and O atoms**. Bader charge of (a) RuO_2, (b) $W_{0.2}Ru_{0.8}O_{2-\delta}$, (c) $Er_{0.1}Ru_{0.9}O_{2-\delta}$, (d) $W_{0.2}Er_{0.1}Ru_{0.7}O_{2-\delta}$ (Red: O; pink: Ir; yellow: Er; light blue: W).

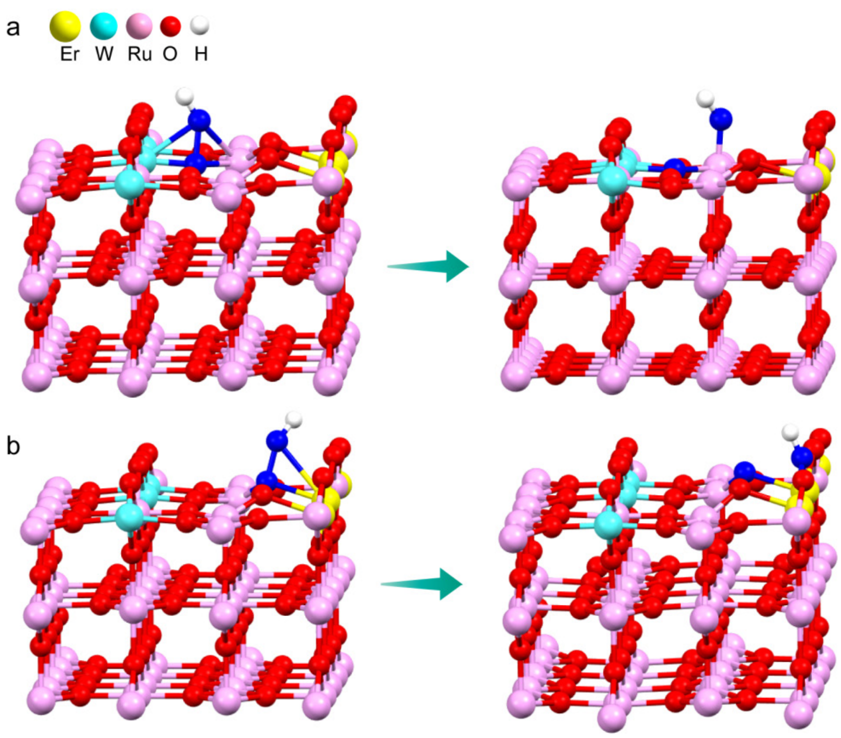

Supplementary Figure 3. **The lattice oxygen oxidation simulation**. (a) The simulation of the oxygen between W and Ru participating in the reaction. (b) The simulation of the oxygen between Er and Ru participating in the reaction.

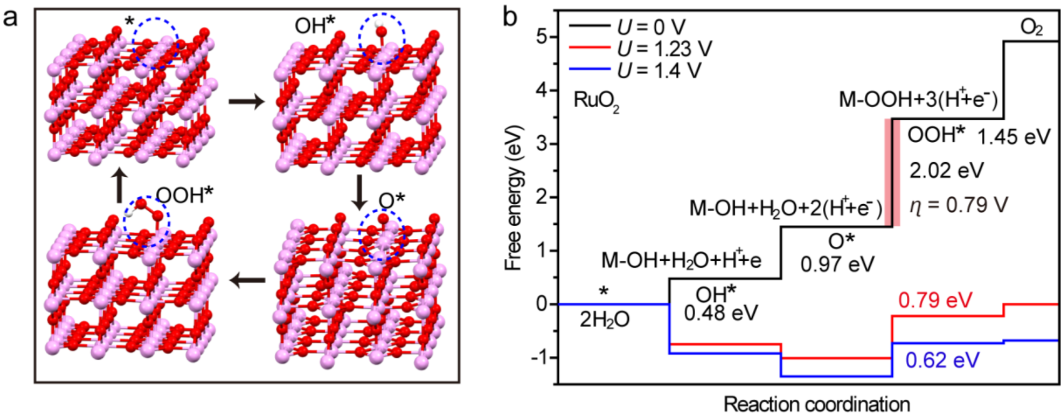

Supplementary Figure 4. **DFT calculation for RuO_2**. (a) Theoretical calculations of acidic OER on the established model of RuO_2. (b) The Free energies of RuO_2 calculated at U=0, 1.23, 1.4 V, respectively. The thick line in the figure's caption means PDS (Red: O; pink: Ir; white: H).

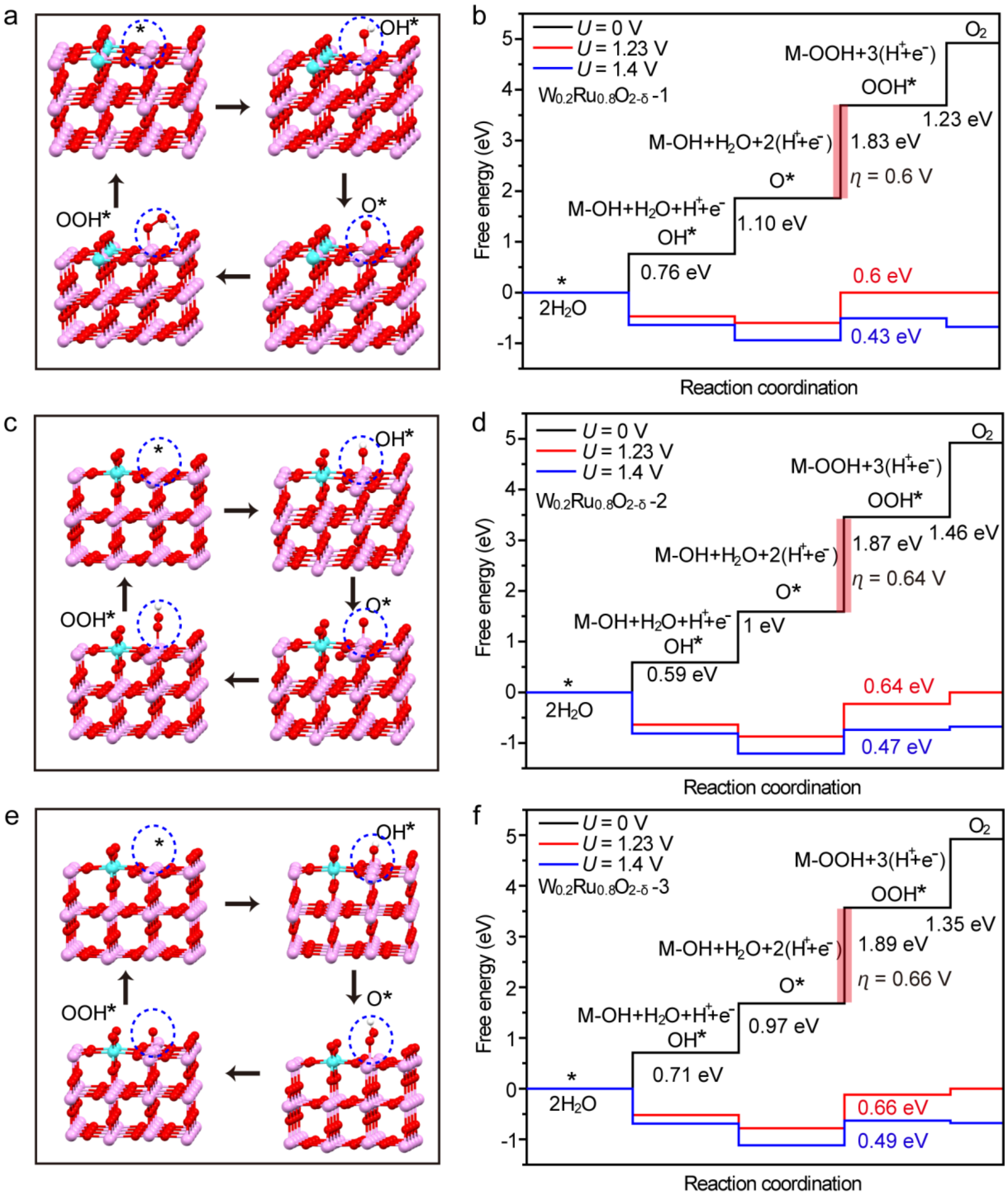

Supplementary Figure 5. DFT calculation for different loacions of Ru active sites in $W_{0.2}Ru_{0.8}O_{2-\delta}$. Theoretical calculations of acidic OER on the established model of (a) $W_{0.2}Ru_{0.8}O_{2-\delta}$-1, (c) $W_{0.2}Ru_{0.8}O_{2-\delta}$-1, (e) $W_{0.2}Ru_{0.8}O_{2-\delta}$-3. The Free energies of (b) $W_{0.2}Ru_{0.8}O_{2-\delta}$-1, (d) $W_{0.2}Ru_{0.8}O_{2-\delta}$-1, (f) $W_{0.2}Ru_{0.8}O_{2-\delta}$-3 calculated at U=0, 1.23, 1.4 V, respectively. The thick line in the figure's caption means PDS (The number 1, 2, 3 represents the various established models with various doping locations in RuO_2, Red: O; pink: Ir; light blue: W; white: H).

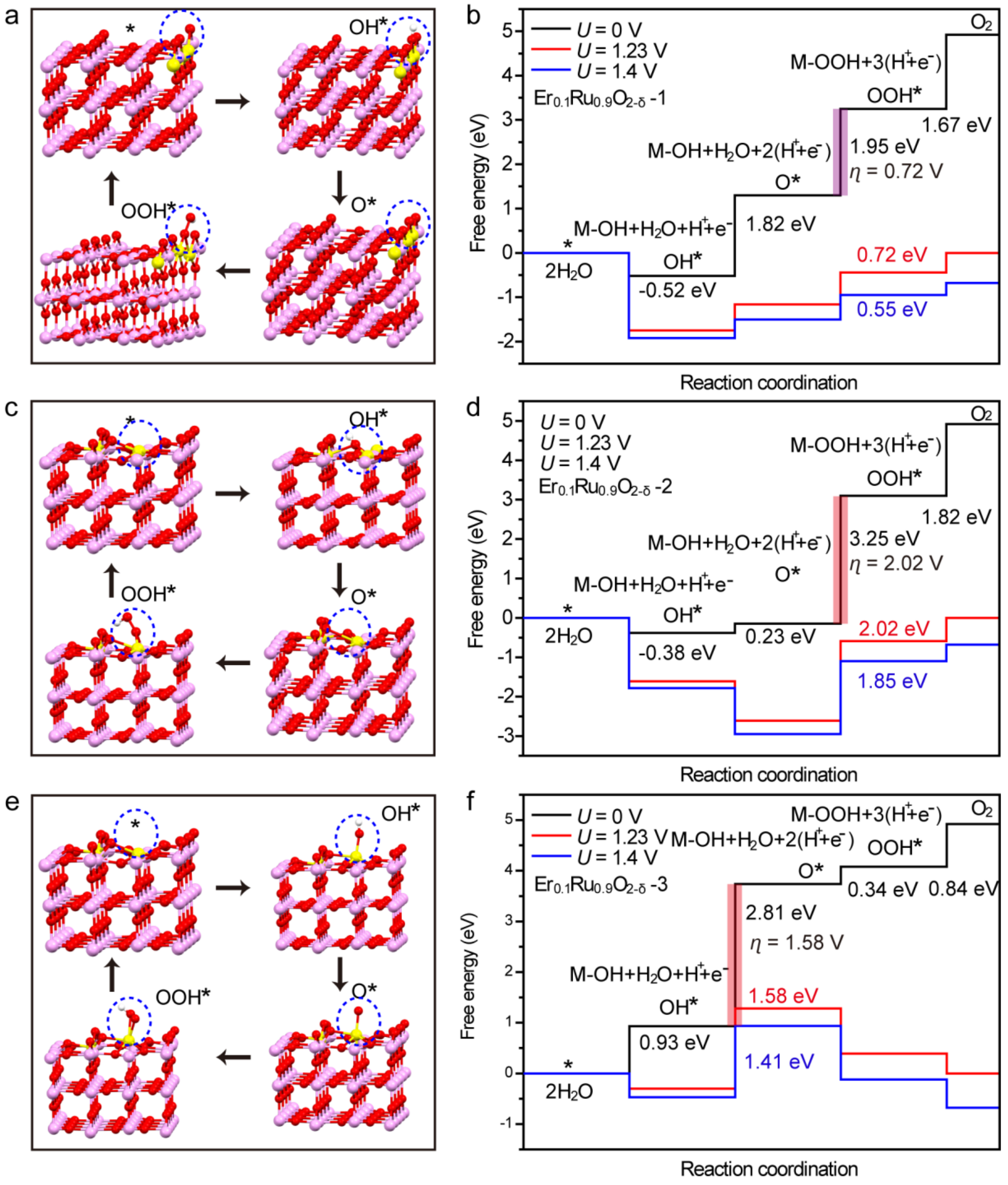

Supplementary Figure 6. DFT calculation for different loacions of Ru active sites in $Er_{0.1}Ru_{0.9}O_{2-\delta}$. Theoretical calculations of acidic OER on the established model of (a) $Er_{0.1}Ru_{0.9}O_{2-\delta}$-1, (c) $Er_{0.1}Ru_{0.9}O_{2-\delta}$-2, (e) $Er_{0.1}Ru_{0.9}O_{2-\delta}$-3. The Free energies of (b) $Er_{0.1}Ru_{0.9}O_{2-\delta}$-1, (d) $Er_{0.1}Ru_{0.9}O_{2-\delta}$-2, (f) $Er_{0.1}Ru_{0.9}O_{2-\delta}$-3 calculated at U=0, 1.23, 1.4 V, respectively. The thick line in the figure's caption means PDS. (The number 1, 2, 3 represents the various established models with various doping locations in RuO_2, Red: O; pink: Ir; yellow: Er; white: H).

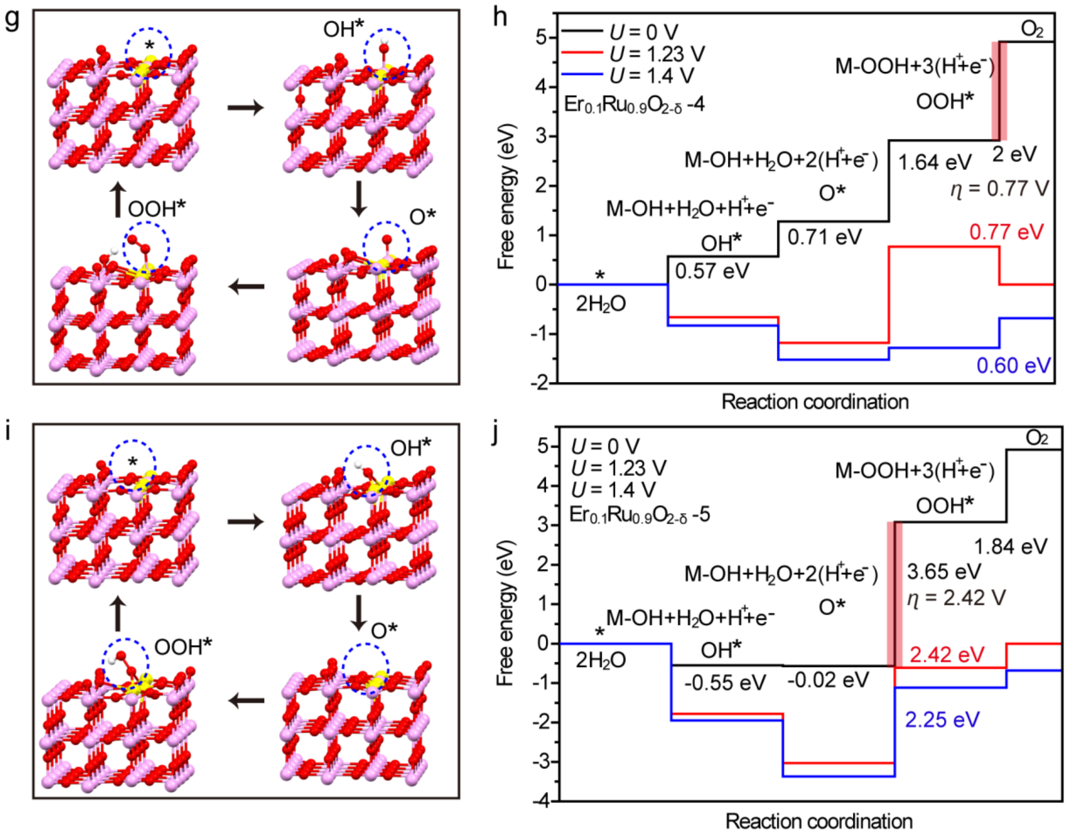

Supplementary Figure 7. **DFT calculation for different loacions of Ru active sites in $Er_{0.1}Ru_{0.9}O_{2-\delta}$.** Theoretical calculations of acidic OER on the established model of (g) $Er_{0.1}Ru_{0.9}O_{2-\delta}$-5 and (i) $Er_{0.1}Ru_{0.9}O_{2-\delta}$-6. The Free energies of (h) $Er_{0.1}Ru_{0.9}O_{2-\delta}$-5 and (j) $Er_{0.1}Ru_{0.9}O_{2-\delta}$-6 calculated at U=0, 1.23, 1.4 V, respectively. The thick line in the figure's caption means PDS. (The number 4 and 5 represent the various established models with various doping locations in RuO_2, Red: O; pink: Ir; yellow: Er; white: H).

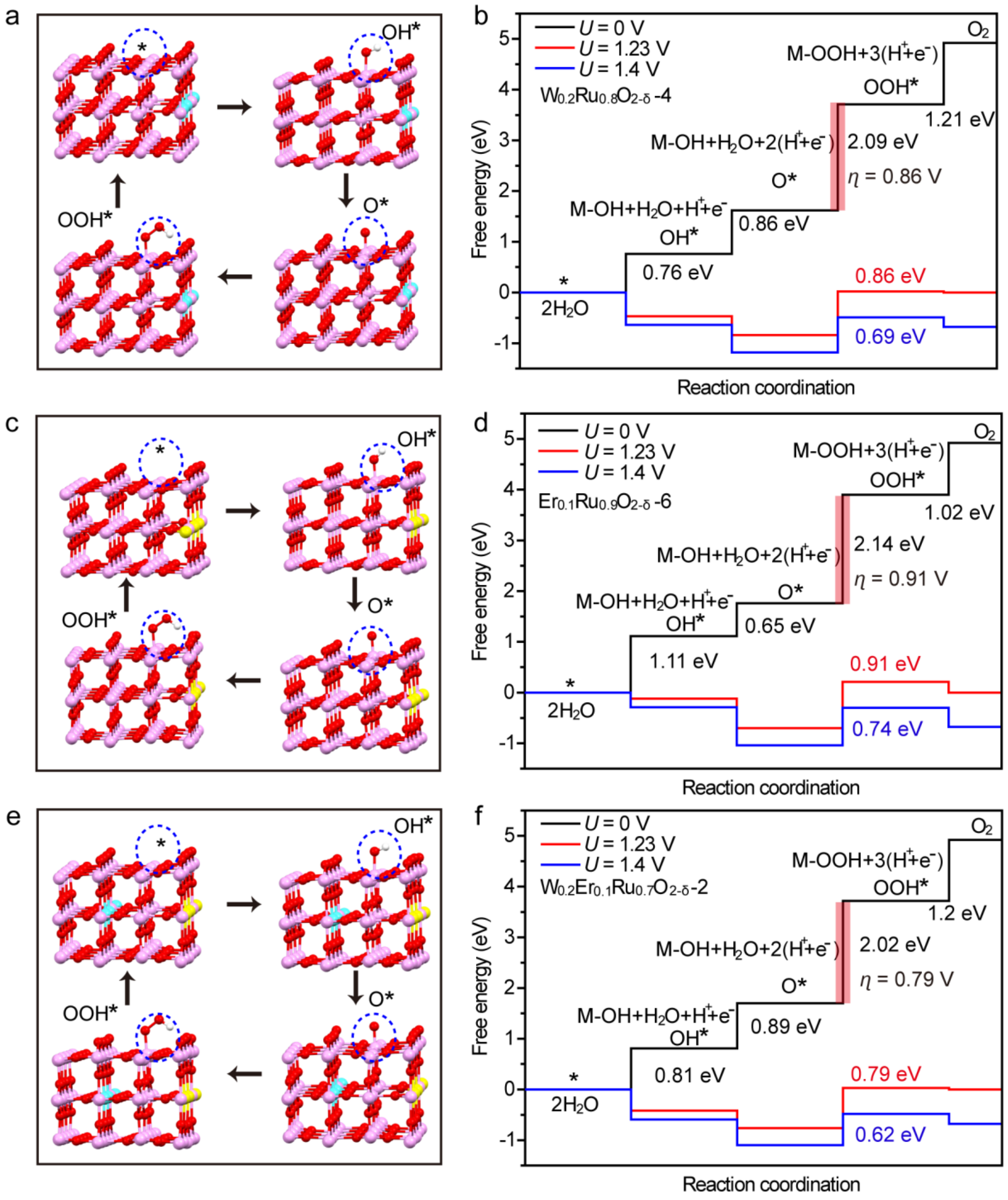

Supplementary Figure 8. **DFT calculation for W and Er in the second layer of RuO_2**. Theoretical calculations of acidic OER on the established model of (a) $W_{0.2}Ru_{0.8}O_{2-\delta}$-4, (c) $Er_{0.1}Ru_{0.9}O_{2-\delta}$-6, (e) $W_{0.2}Er_{0.1}Ru_{0.7}O_{2-\delta}$-2, respectively. The Free energies of (b) $W_{0.2}Ru_{0.8}O_{2-\delta}$-4, (d) $Er_{0.1}Ru_{0.9}O_{2-\delta}$-6, (f) $W_{0.2}Er_{0.1}Ru_{0.7}O_{2-\delta}$-2 calculated at U=0, 1.23, 1.4 V, respectively. The thick line in the figure's caption means PDS. (The number 2, 4, 6 represent the various established models with various doping locations in RuO_2, Red: O; pink: Ir; yellow: Er; light blue: W; white: H).

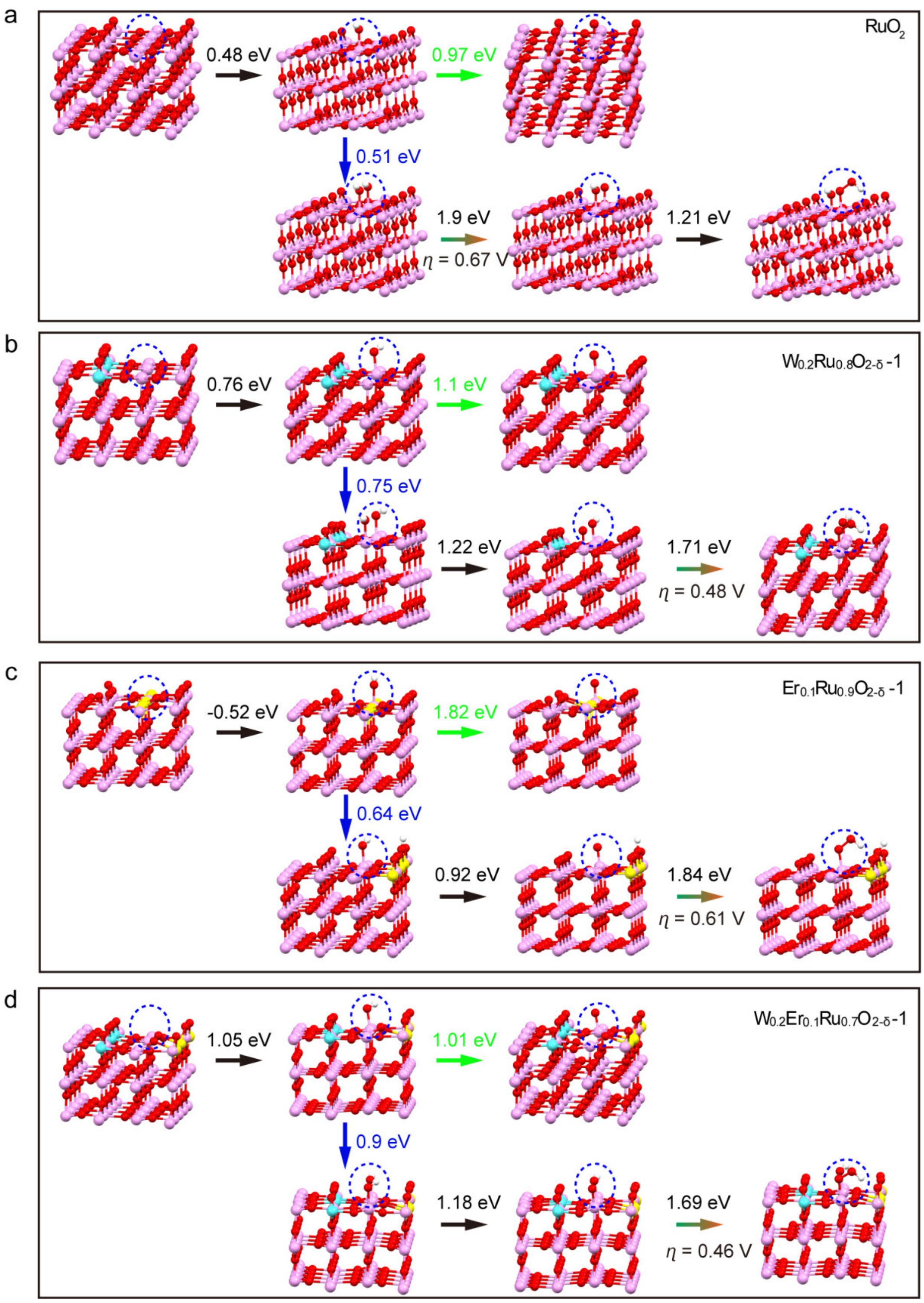

Supplementary Figure 9. **DFT calculation of the neighboring intermediates on the energetics**. Theoretical calculations of the neighboring intermediates on the energetics for the established models (a) RuO_2, (b) $W_{0.2}Ru_{0.8}O_{2-\delta}$-1, (c) $Er_{0.1}Ru_{0.9}O_{2-\delta}$-1, and (d) $W_{0.2}Er_{0.1}Ru_{0.7}O_{2-\delta}$-1, respectively (Red: O; pink: Ir; yellow: Er; light blue: W; white: H).

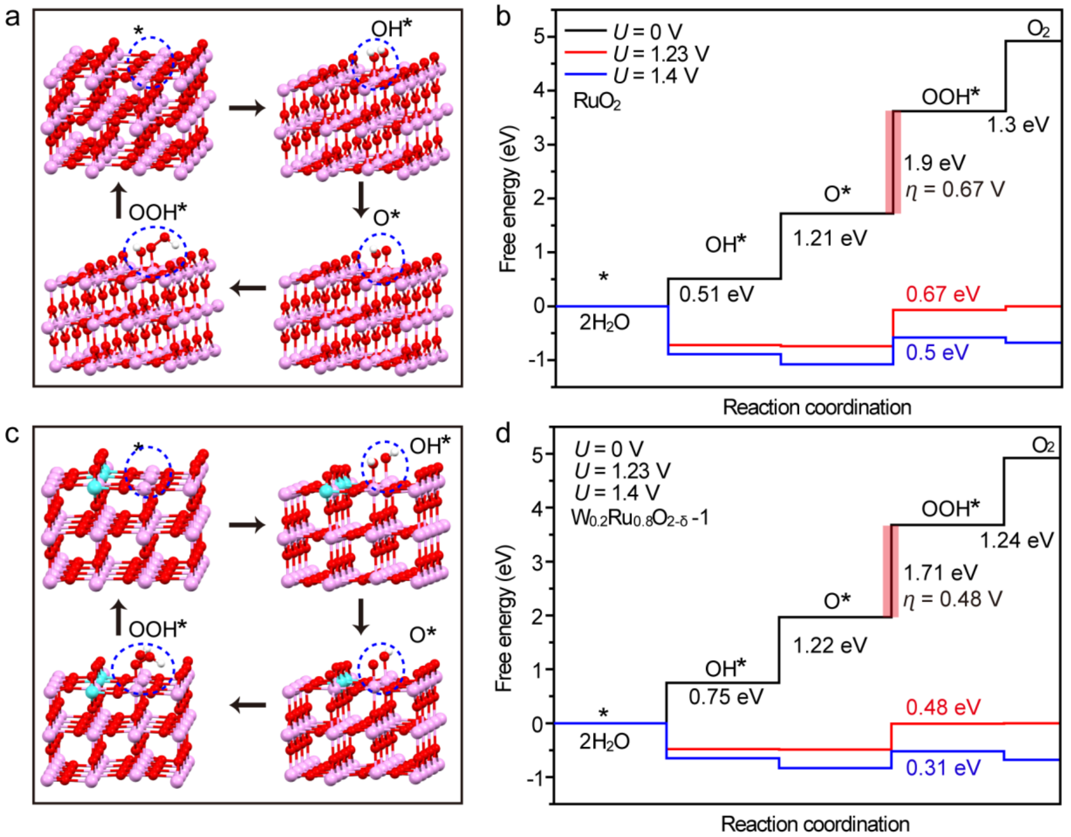

Supplementary Figure 10. **DFT calculation of the neighboring intermediates on the energetics**. The neighboring intermediates on the established models (a) RuO_2, (c) $W_{0.2}Ru_{0.8}O_{2-\delta}$-1. Theoretical calculations of the neighboring intermediates on the energetics for the established models (b) RuO_2, (d) $W_{0.2}Ru_{0.8}O_{2-\delta}$-1 at U=0, 1.23, 1.4 V, respectively. The thick line in the figure's caption means PDS. (Red: O; pink: Ir; light blue: W; white: H).

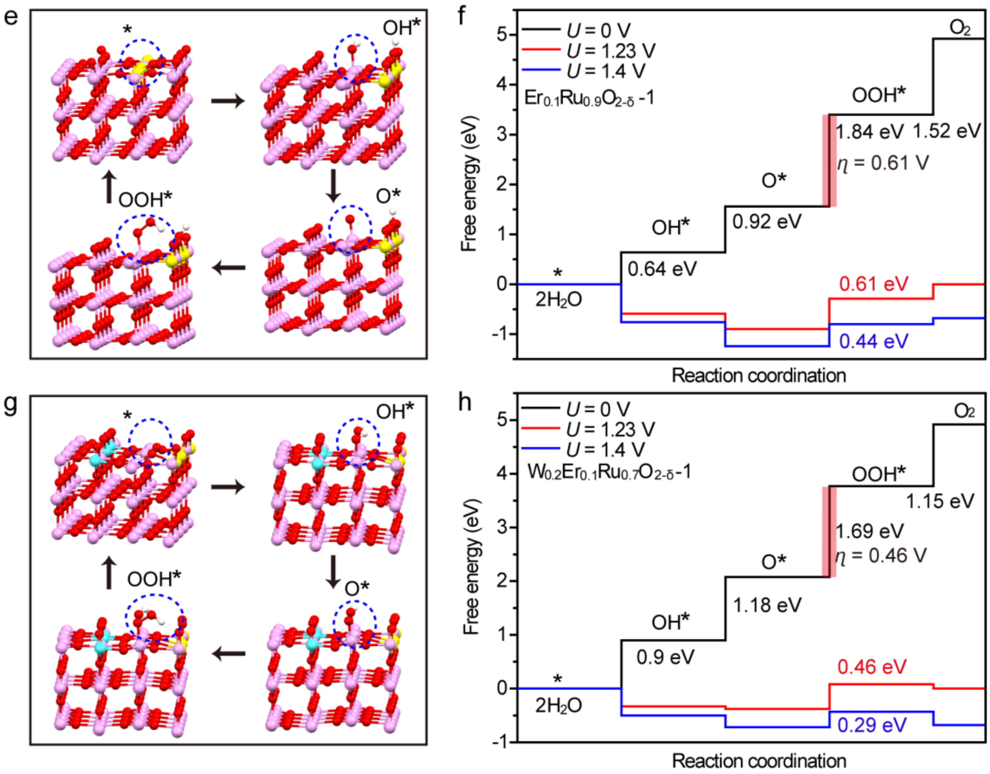

Supplementary Figure 11. **DFT calculation of the neighboring intermediates on the energetics**. The neighboring intermediates on the established models (a) $Er_{0.1}Ru_{0.9}O_{2-\delta}$-1, (c) $W_{0.2}Er_{0.1}Ru_{0.7}O_{2-\delta}$-1. Theoretical calculations of the neighboring intermediates on the energetics for the established models (b) $Er_{0.1}Ru_{0.9}O_{2-\delta}$-1, (d) $W_{0.2}Er_{0.1}Ru_{0.7}O_{2-\delta}$-1 at U=0, 1.23, 1.4 V, respectively. The thick line in the figure's caption means PDS. (Red: O; pink: Ir; yellow: Er; light blue: W; white: H).

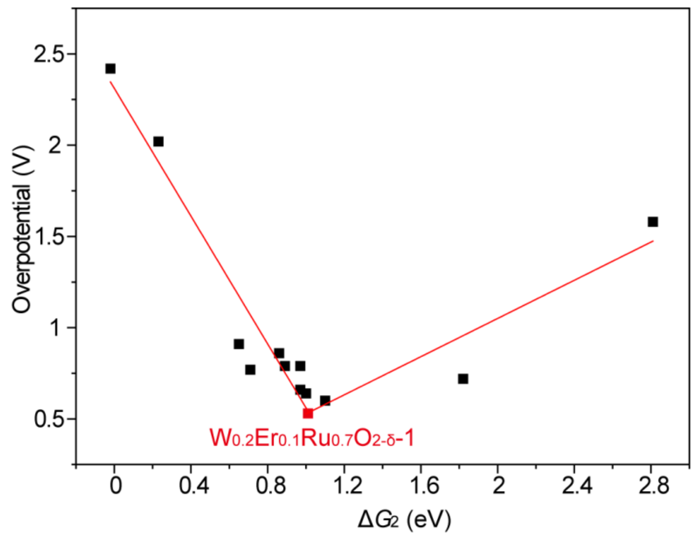

Supplementary Figure 12. **Volcano relationship between overpotential and ΔG_2**. Overpotential-ΔG_2 volcano relation for these established structures toward OER. The other symbols represent the other established models.

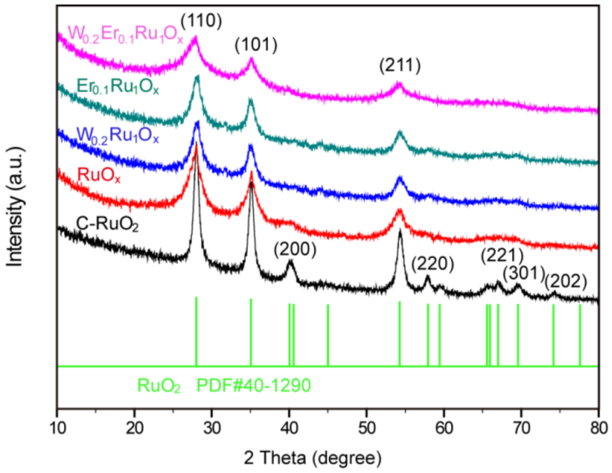

Supplementary Figure 13. **XRD patterns for these samples**. XRD patterns for C-RuO_2 and the synthesized $RuO_{2-\delta}$, $W_{0.2}Ru_{0.8}O_{2-\delta}$, $Er_{0.1}Ru_{0.9}O_{2-\delta}$, and $W_{0.2}Er_{0.1}Ru_{0.7}O_{2-\delta}$ nanosheets.

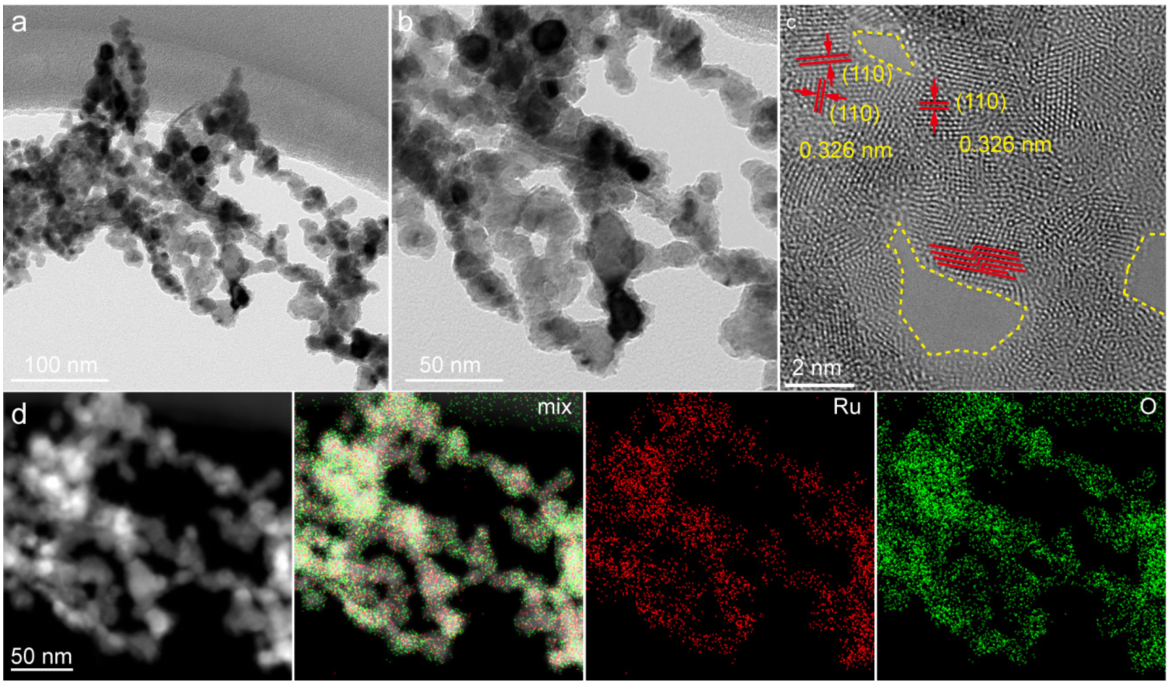

Supplementary Figure 14. **TEM characterization of $RuO_{2-\delta}$**. (a) TEM image, (b) high-magnification TEM image, (c) HR-TEM image, (d) HAADF-TEM image and elemental maps for $RuO_{2-\delta}$ nanosheets.

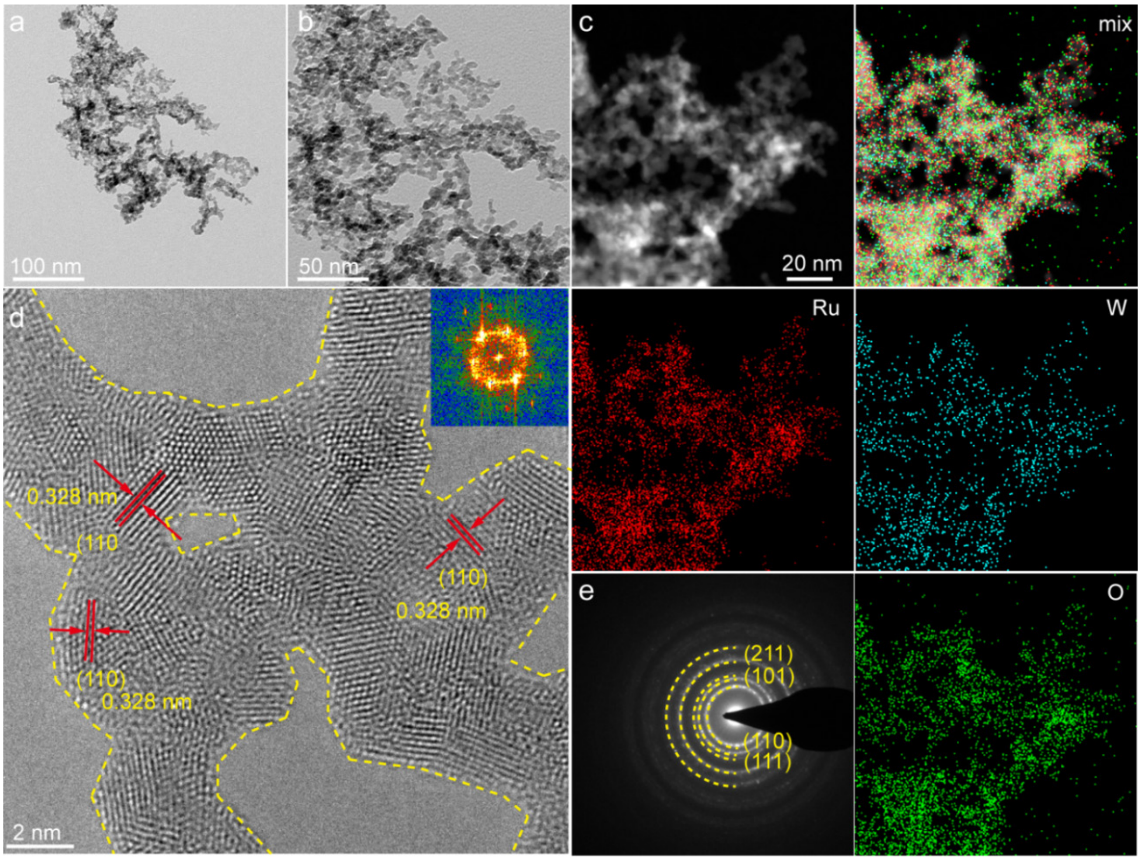

Supplementary Figure 15. **TEM characterization of $W_{0.1}Ru_{0.9}O_{2-\delta}$.** (a) TEM image, (b) high-magnification TEM image, (c) HAADF-TEM image and elemental maps, (d) HR-TEM image, (e) SAED image for $W_{0.1}Ru_{0.9}O_{2-\delta}$ nanosheets.

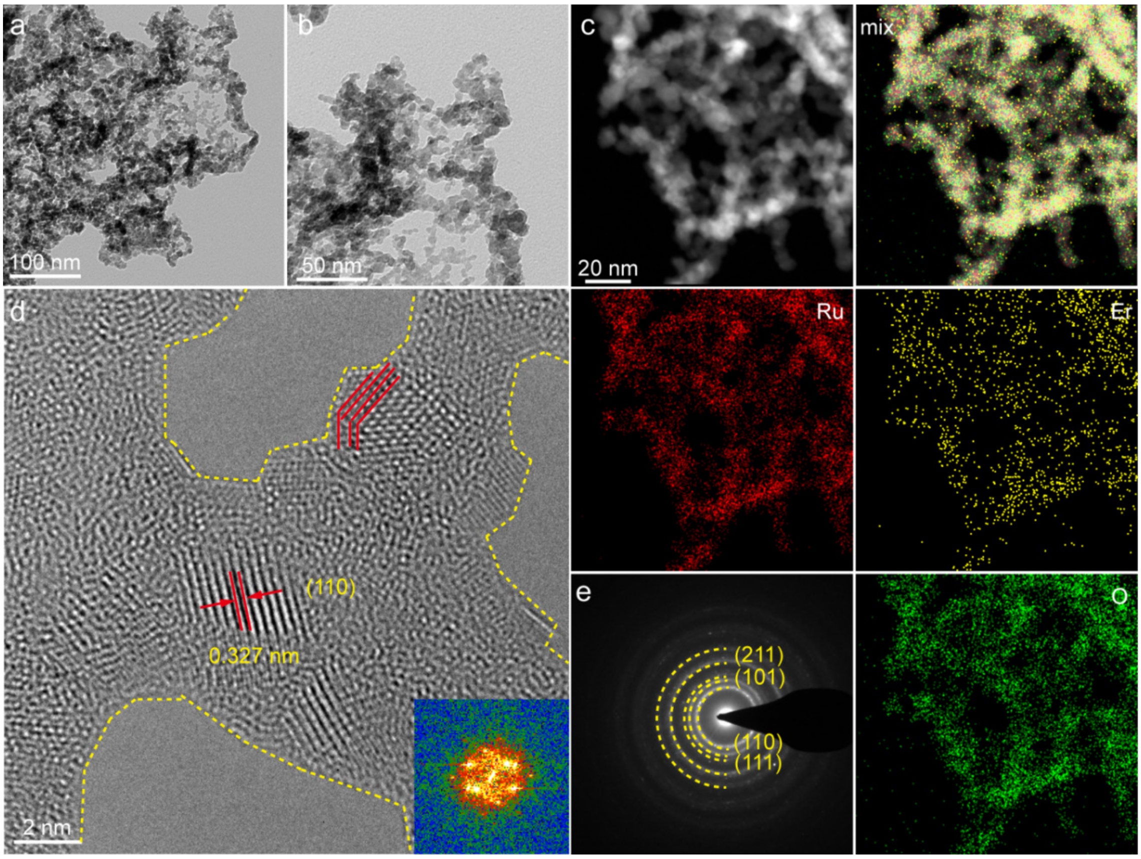

Supplementary Figure 16. **TEM characterization of $Er_{0.1}Ru_{0.9}O_{2-\delta}$.** (a) TEM image, (b) high-magnification TEM image, (c) HAADF-TEM image and elemental maps, (d) HR-TEM image, (e) SAED image for $Er_{0.1}Ru_{0.9}O_{2-\delta}$ nanosheets.

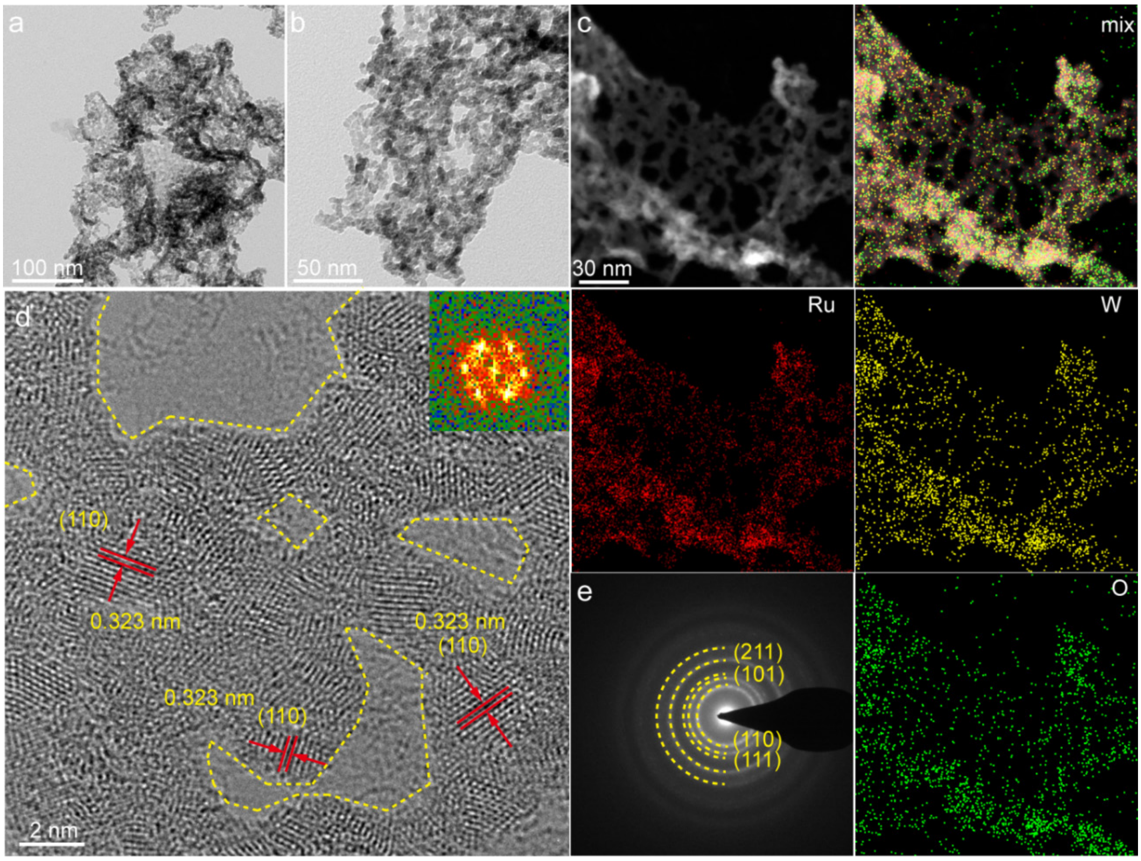

Supplementary Figure 17. **TEM characterization of $W_{0.2}Ru_{0.8}O_{2-\delta}$**. (a) TEM image, (b) high-magnification TEM image, (c) HAADF-TEM image and elemental maps, (d) HR-TEM image, (e) SAED image for $W_{0.2}Ru_{0.8}O_{2-\delta}$ nanosheets.

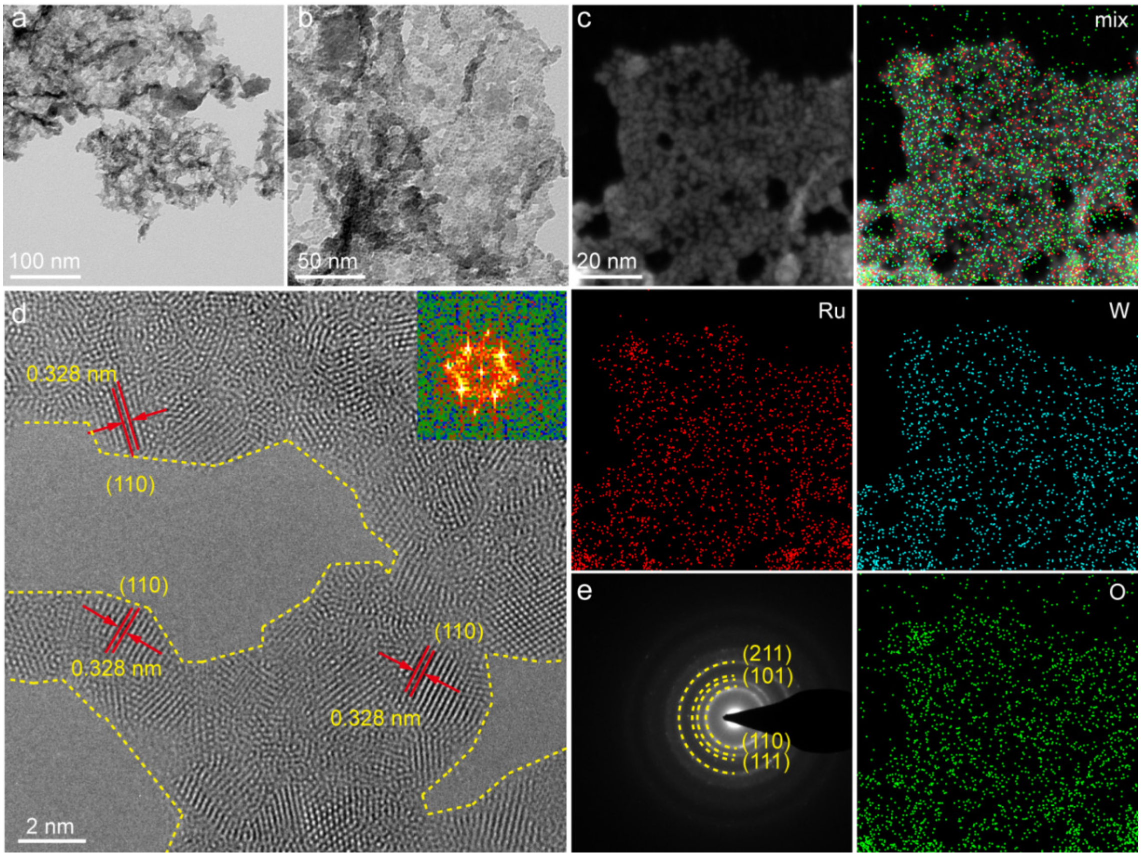

Supplementary Figure 18. **TEM characterization of $W_{0.3}Ru_{0.7}O_{2-\delta}$**. (a) TEM image, (b) high-magnification TEM image, (c) HAADF-TEM image and elemental maps, (d) HR-TEM image, (e) SAED image for $W_{0.3}Ru_{0.7}O_{2-\delta}$ nanosheets.

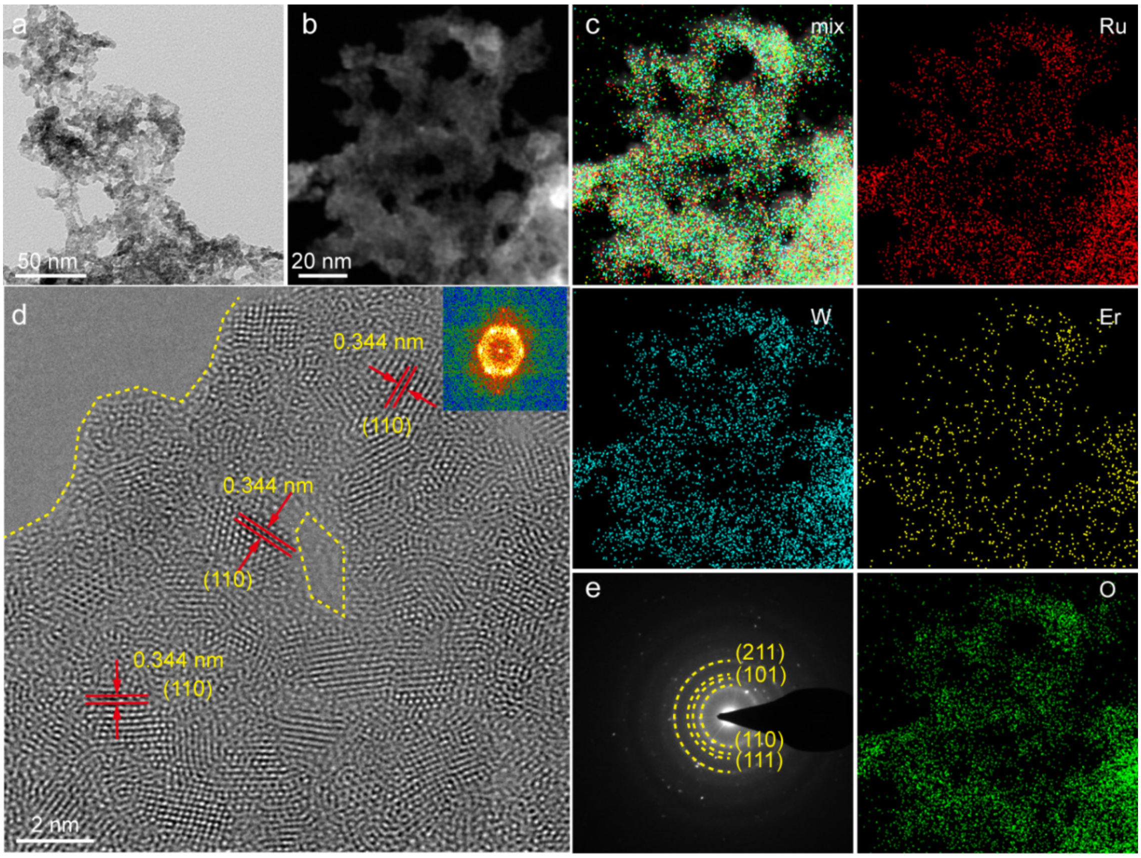

Supplementary Figure 19. TEM characterization of $W_{0.3}Ru_{0.7}O_{2-\delta}$. (a) TEM image, (b) high-magnification TEM image, (c) HAADF-TEM image and elemental maps, (d) HR-TEM image, (e) SAED image for $W_{0.2}Er_{0.2}Ru_{0.6}O_{2-\delta}$ nanosheets.

Supplementary Table 1. ICP analysis for $W_{0.1}Ru_{0.9}O_{2-\delta}$.

Element	Sample amount	Conversion content	at.%
W	15 mg	3603.3	9.8
Ru	15 mg	18233	90.2

Supplementary Table 2. ICP analysis for $W_{0.2}Ru_{0.8}O_{2-\delta}$.

Element	Sample amount	Conversion content	at.%
W	15 mg	4679.6	16.3
Ru	15 mg	12318.6	82.7

Supplementary Table 3. ICP analysis for $W_{0.3}Ru_{0.7}O_{2-\delta}$.

Element	Sample amount	Conversion content	at.%
W	15 mg	5423.3	29.5
Ru	15 mg	7125.4	70.5

Supplementary Table 4. ICP analysis for $Er_{0.1}Ru_{0.9}O_{2-\delta}$.

Element	Sample amount	Conversion content	at.%
Er	15 mg	2656.2	10.1
Ru	15 mg	14398.6	89.9

Supplementary Table 5. ICP analysis for $W_{0.2}Er_{0.1}Ru_{0.7}O_{2-\delta}$.

Element	Sample amount	Conversion content	at.%
W	15 mg	4079.6	15.5
Er	15 mg	1856.6	7.8
Ru	15 mg	11118.6	76.7

Supplementary Table 6. ICP analysis for $W_{0.2}Er_{0.2}Ru_{0.6}O_{2-\delta}$.

Element	Sample amount	Conversion content	at.%
W	15 mg	7096.2	19.3
Er	15 mg	6389.3	19.1
Ru	15 mg	12451.8	61.6

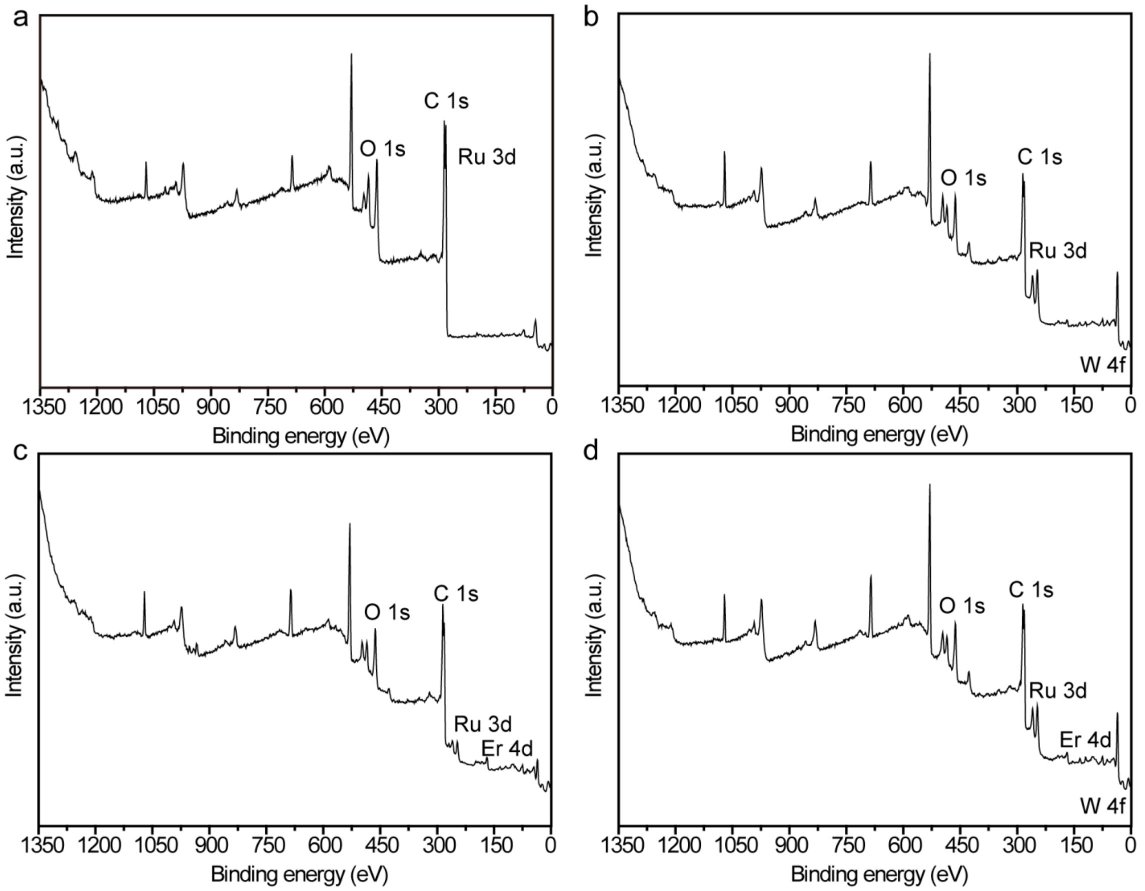

Supplementary Figure 20. **The wide XPS spectra**. The wide XPS spectra for (a) $RuO_{2-\delta}$, (b) $W_{0.2}Ru_{0.8}O_{2-\delta}$, (c) $Er_{0.1}Ru_{0.9}O_{2-\delta}$, and (d) $W_{0.2}Er_{0.1}Ru_{0.7}O_{2-\delta}$ nanosheets.

Supplementary Table 7. EXAFS fitting parameters of Ru K-edge for various samples (S_0^2=0.829).

Sample	Shell	N^a	R(Å)^b	σ^2(Å^2)^c	ΔE_0(eV)^d	R factor
Ru foil	Ru-Ru	12	2.68	0.0037	0.3	0.0094
RuO_2	Ru-O	6.0	1.98	0.0036	-2.5	0.0009
	Ru-Ru	1.8	3.12	0.0013		
	Ru-O	4.2	3.37	0.0036		
	Ru-Ru	3.8	3.56	0.0013		
$RuO_{2-\delta}$	Ru-O	6.0	1.97	0.0037	-3.0	0.0044
	Ru-Ru	1.2	2.68	0.0027		
	Ru-Ru	1.4	3.11	0.0027		
	Ru-O	2.7	3.36	0.0011		
	Ru-Ru	4.2	3.56	0.0027		
$W_{0.2}Er_{0.1}Ru_{0.7}O_{2-\delta}$	Ru-O	5.6	1.97	0.0036	-1.9	0.0026
	Ru-Ru	1.2	2.71	0.0028		
	Ru-Ru	1.5	3.11	0.0025		
	Ru-O	2.9	3.38	0.0016		
	Ru-Ru	2.4	3.56	0.0025		
	Ru-W/Er	1.0	3.55	0.0029		

$^a N$: coordination numbers; $^b R$: bond distance; $^c\sigma^2$: Debye-Waller factors; ^{d} ΔE_0: the inner potential correction. R factor: goodness of fit. S_0^2 was set to 0.829, according to the experimental EXAFS fit of Ru foil reference by fixing CN as the known crystallographic value.

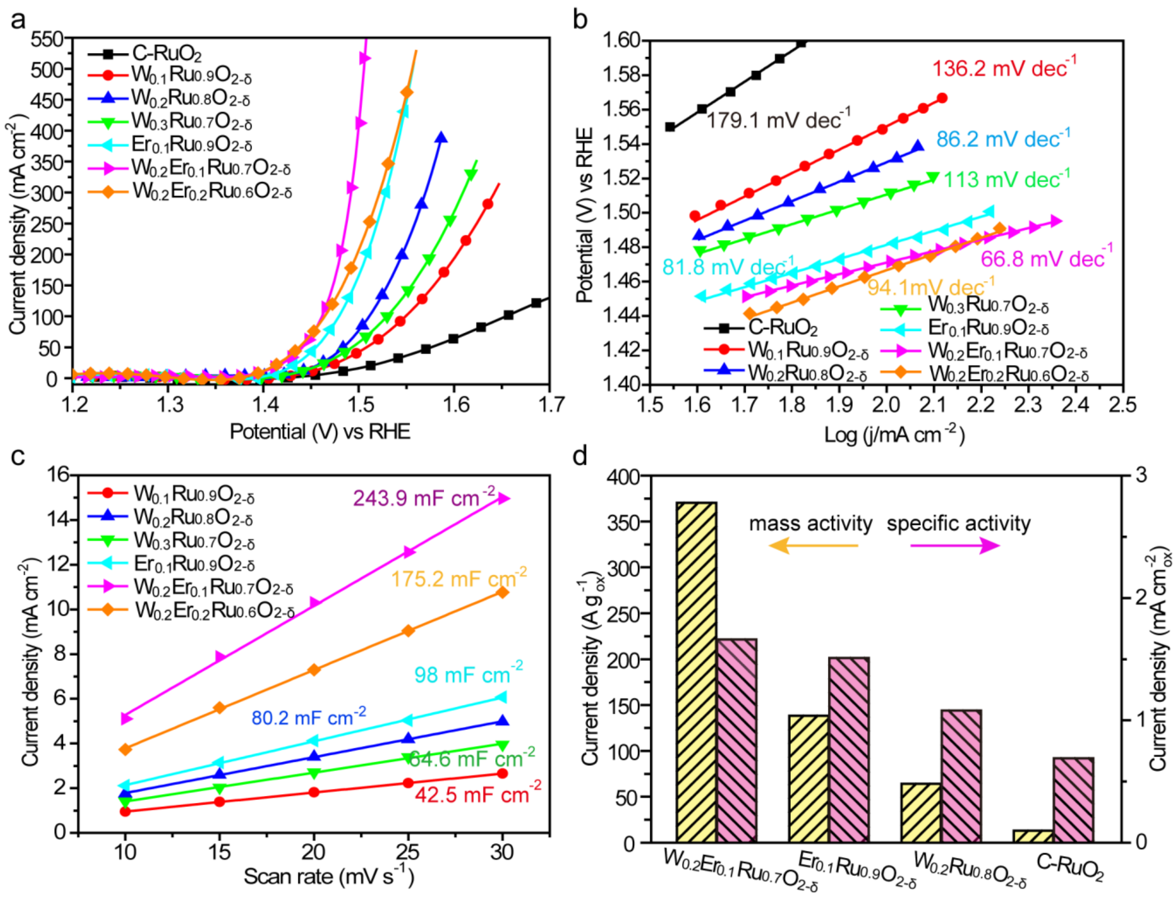

Supplementary Figure 21. **Electrocatalytic measurement for these samples**. (a) Polarization curves, (b) Corresponding Tafel slopes calculated from (a), (c) C_{dl} plots inferred from CV curves for C-RuO_2, RuO_2, $W_{0.1}Ru_{0.9}O_{2-\delta}$, $W_{0.2}Ru_{0.8}O_{2-\delta}$, $W_{0.3}Ru_{0.7}O_{2-\delta}$, $Er_{0.1}Ru_{0.9}O_{2-\delta}$, $W_{0.2}Er_{0.1}Ru_{0.7}O_{2-\delta}$, and $W_{0.2}Er_{0.2}Ru_{0.6}O_{2-\delta}$ nanosheets, (d) Mass and specific activities were compared at η = 275 mV for C-RuO_2, $W_{0.1}Ru_{0.9}O_{2-\delta}$, $Er_{0.1}Ru_{0.9}O_{2-\delta}$, and $W_{0.2}Er_{0.1}Ru_{0.7}O_{2-\delta}$ nanosheets applying in 0.5 M H_2SO_4 electrolyte.

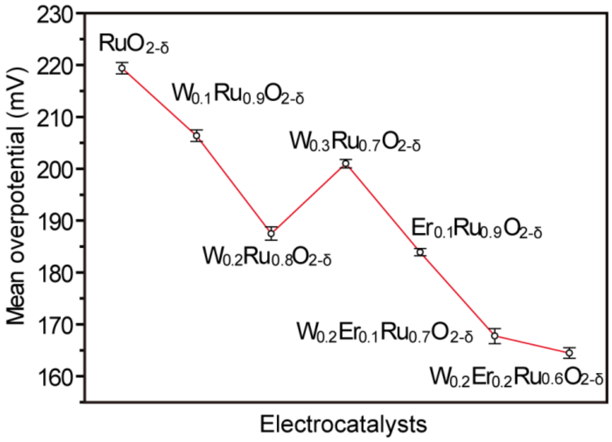

Supplementary Figure 22. **The error bars after 500th LSV curves of each sample**. The overpotentials at 10 mA cm^{-2} from repeated LSV curves for these prepared materials. The overpotentials of 100th, 300th, and 500th LSV curves minuses the overpotential of the 1st LSV curve at 10 mA·cm^{-2}. Then, the average of the difference was considered as the error.

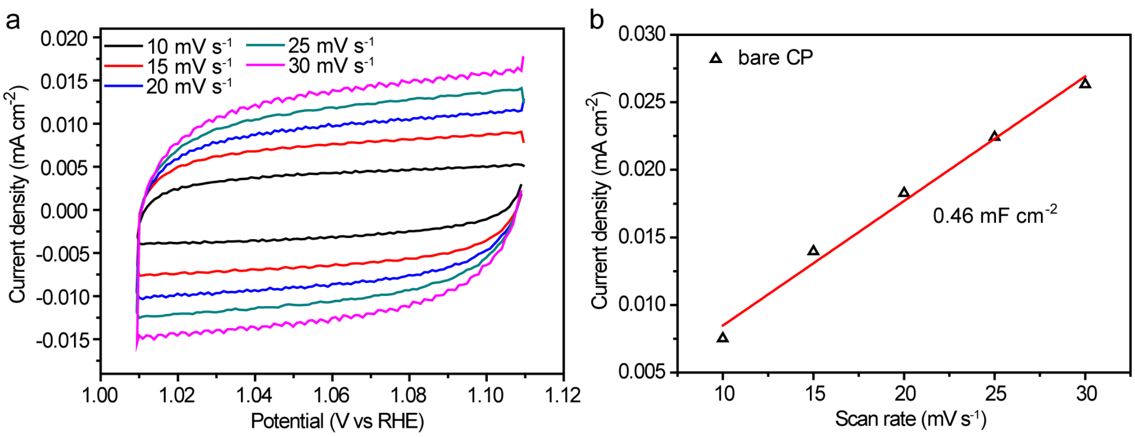

Supplementary Figure 23. **CV for bare CP**. (a) CV curves of the bare CP, (b) the calculated C_{dl} from CV curves.

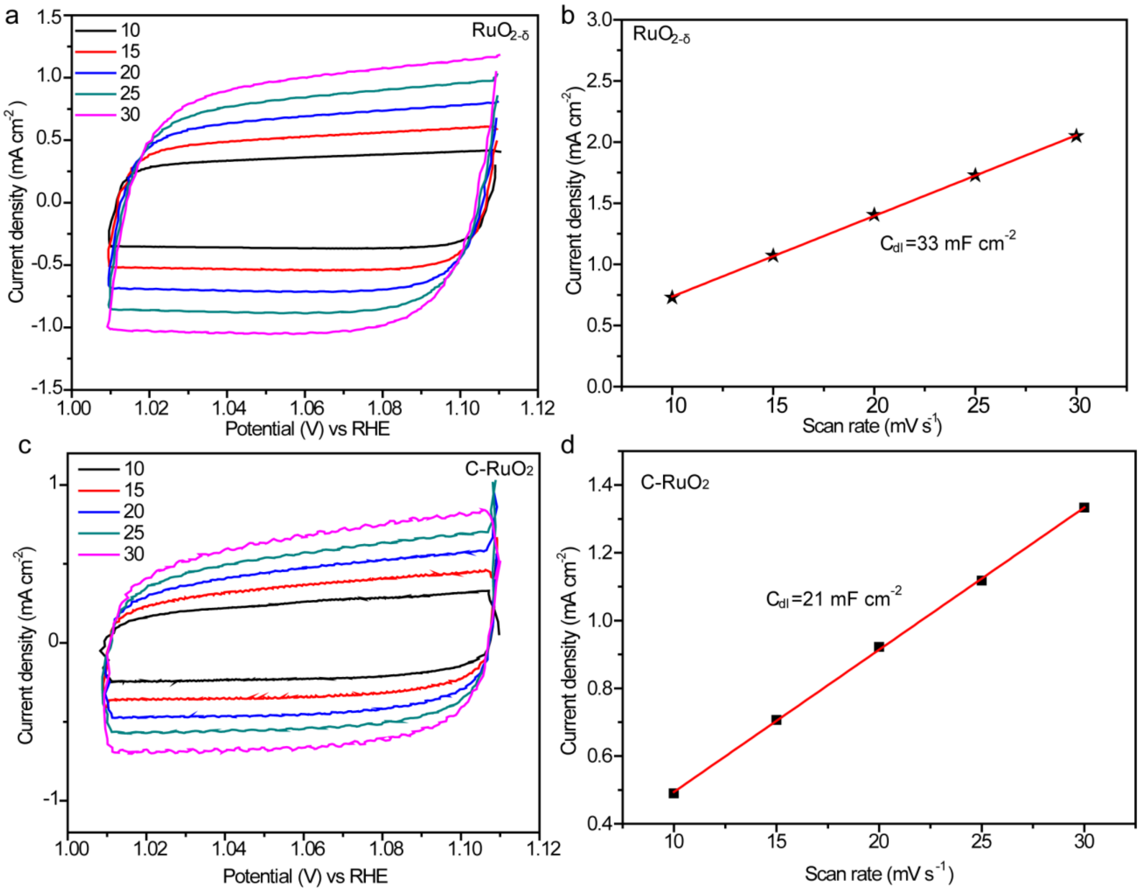

Supplementary Figure 24. **CV and calculated C_{dl} for $RuO_{2-\delta}$ and C-RuO_2**.Non-Faradaic scan for double-layer capacitance. Cyclic voltammetry of (a) the prepared $RuO_{2-\delta}$ nanosheets, (b) C_{dl} of the prepared $RuO_{2-\delta}$ calculated from (a), (c) CV curves for C-RuO_2, and (d) C_{dl} of C-RuO_2 calculated from (c).

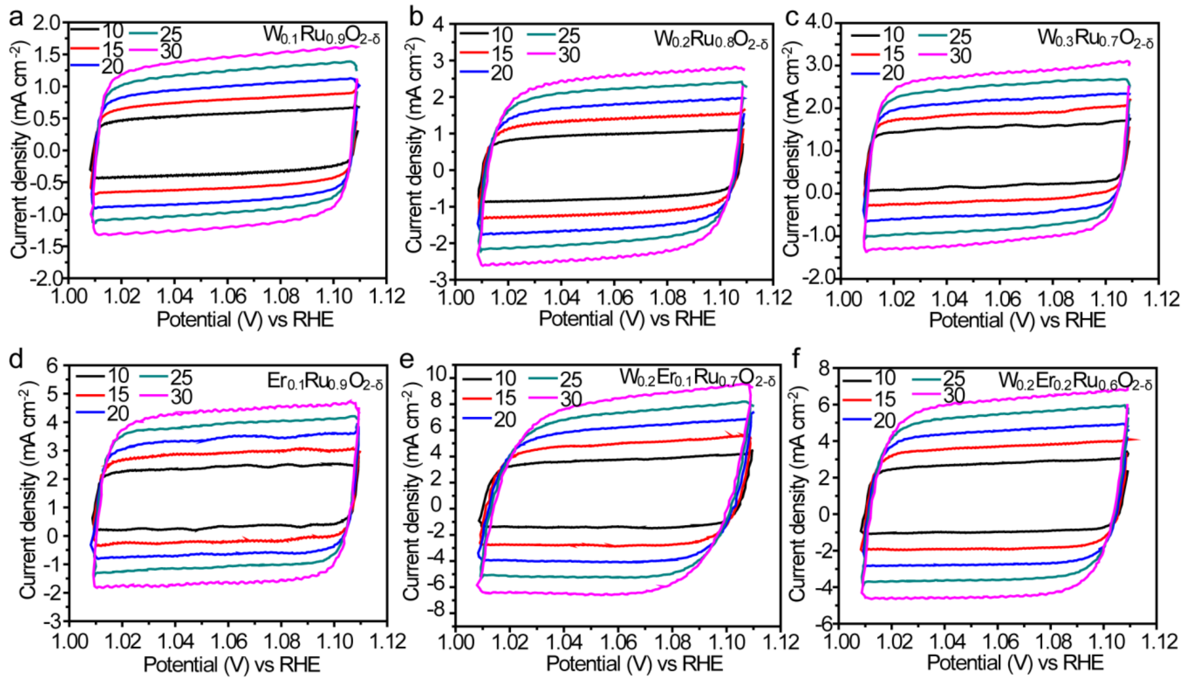

Supplementary Figure 25. **CV curves for these prepared samples on CP**. Non-Faradaic scan for double-layer capacitance. CV curves (Scan rate: mV·s^{-1}) of (a) $W_{0.1}Ru_{0.9}O_{2-\delta}$, (b) $W_{0.2}Ru_{0.8}O_{2-\delta}$, (c) $W_{0.3}Ru_{0.7}O_{2-\delta}$, (d) $Er_{0.1}Ru_{0.9}O_{2-\delta}$, (e) $W_{0.2}Er_{0.1}Ru_{0.7}O_{2-\delta}$, and (f) $W_{0.2}Er_{0.2}Ru_{0.6}O_{2-\delta}$ nanosheets.

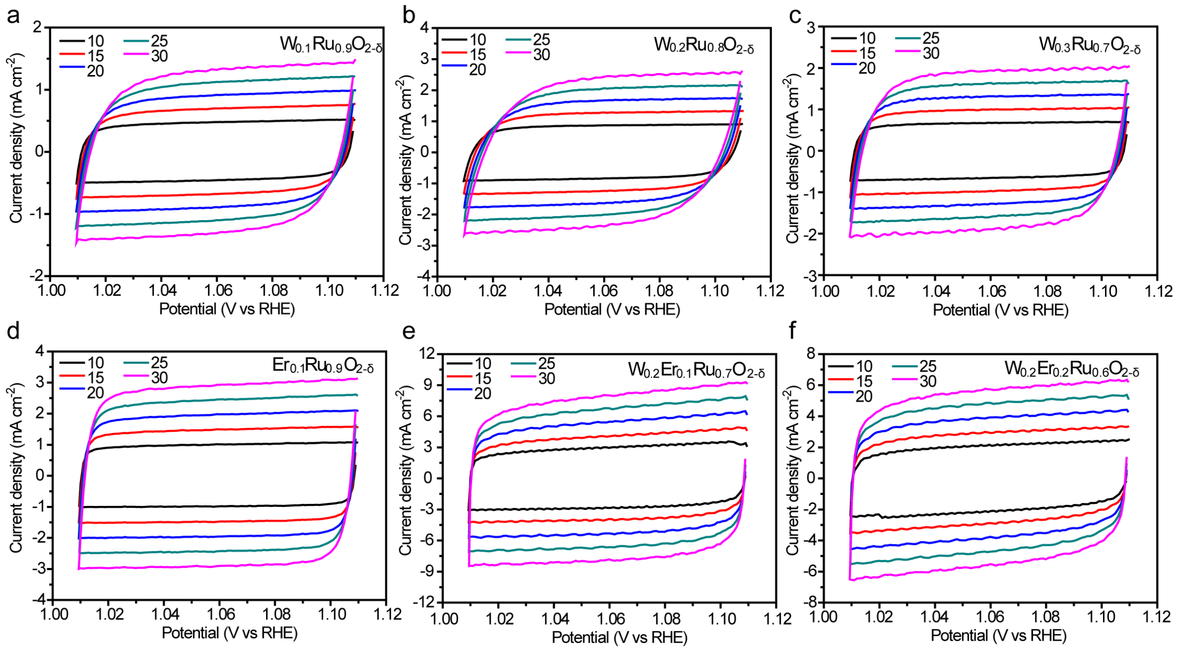

Supplementary Figure 26. **CV curves for these prepared samples on GC**. Non-Faradaic scan for double-layer capacitance. CV curves (Scan rate: $mV{\cdot}s^{-1}$) of (a) $W_{0.1}Ru_{0.9}O_{2-\delta}$, (b) $W_{0.2}Ru_{0.8}O_{2-\delta}$, (c) $W_{0.3}Ru_{0.7}O_{2-\delta}$, (d) $Er_{0.1}Ru_{0.9}O_{2-\delta}$, (e) $W_{0.2}Er_{0.1}Ru_{0.7}O_{2-\delta}$, and (f) $W_{0.2}Er_{0.2}Ru_{0.6}O_{2-\delta}$ nanosheets loaded on glassy carbon (GC) electrodes.

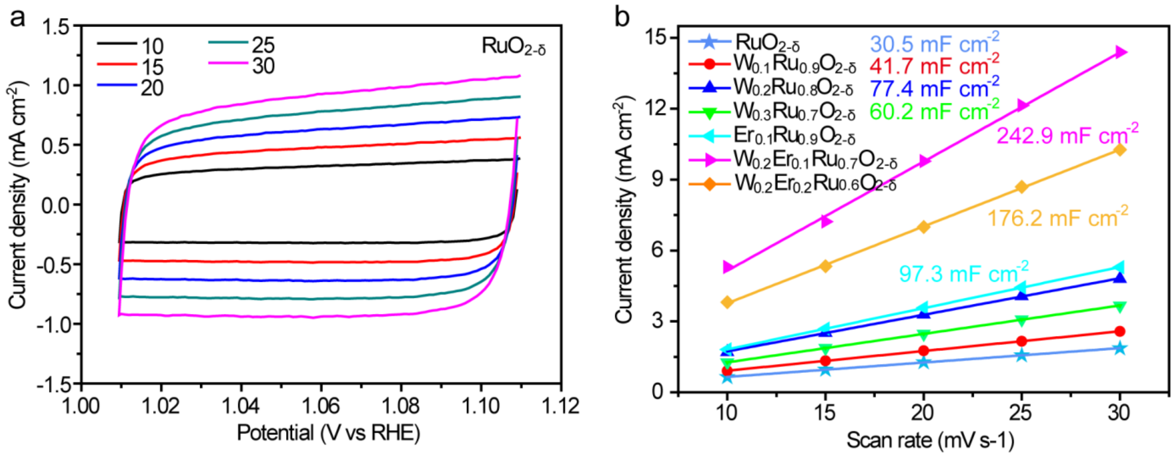

Supplementary Figure 27. **CV curves for $RuO_{2-\delta}$ on CP**. Non-Faradaic scan for double-layer capacitance. (a) CV curves (Scan rate: $mV \cdot s^{-1}$) of $RuO_{2-\delta}$ loading on glassy carbon (GC) electrodes, (b) C_{dl} for $RuO_{2-\delta}$, $W_{0.2}Ru_{0.8}O_{2-\delta}$, $W_{0.3}Ru_{0.7}O_{2-\delta}$, $Er_{0.1}Ru_{0.9}O_{2-\delta}$, $W_{0.2}Er_{0.1}Ru_{0.7}O_{2-\delta}$, and $W_{0.2}Er_{0.2}Ru_{0.6}O_{2-\delta}$ nanosheets loading on glassy carbon (GC) electrodes.

Supplementary Table 8. Comparison of the surface area, mass activity, and specific activity for the prepared electrocatalysts with C-RuO_2.

Samples	C_{dl} (mF·cm^{-2})	R_f	Surface area ($m^2 \cdot g^{-1}$)	Mass activity ($A \cdot g^{-1}{}_{ox}$)	Specific activity ($mA \cdot cm^{-2}{}_{ox}$)
$W_{0.2}Er_{0.1}Ru_{0.7}O_{2-\delta}$	243.9	4065	1231.8	1518.6	1.23
$W_{0.2}Er_{0.2}Ru_{0.6}O_{2-\delta}$	175.2	2920	884.8	688.8	0.78
$Er_{0.1}Ru_{0.9}O_{2-\delta}$	98	1633.3	494.9	567.4	1.15
$W_{0.2}Ru_{0.8}O_{2-\delta}$	64.6	1076.7	326.3	377.9	1.16
$W_{0.3}Ru_{0.7}O_{2-\delta}$	80.2	1336.7	405.1	268.24	0.66
$W_{0.1}Ru_{0.9}O_{2-\delta}$	42.5	708.3	214.6	187.5	0.78
RuO_2	33	550	166.7	164.2	0.98
C-RuO_2	21	350	106.1	53.3	0.50

R_f was calculated by C_{dl} dividing the capacitance of these electrocatalysts with smooth surface (0.06 mF cm^{-2}). Surface area was calculated via R_f multiplying the electrode geometrical area and normalized by the loading mass of electrocatalysts.

The mass and specific activities were obtained from the current densities at η = 275 mV.

Supplementary Table 9. Comparison of some representative OER catalysts reported under acidic conditions.

Catalysts	Electrolyte solution	Stability (h)	Overpotentials at the corresponding *j*	Reference
Co-RuIr	0.1 M $HClO_4$	25	η_{10} = 235 mV	1
$CaCu_3Ru_4O_{12}$	0.5 M H_2SO_4	24	η_{10} = 171 mV	2
$Cr_{0.6}Ru_{0.4}O_2$	0.5 M H_2SO_4	10	η_{10} = 178 mV	3
Ru_1-N_4	0.5 M H_2SO_4	30	η_{10} = 267 mV	4
Ru_1-Pt_3Cu	0.1 M $HClO_4$	28	η_{10} = 220 mV	5
Cu-doped RuO_2	0.5 M H_2SO_4	8	η_{10} = 188 mV	6
$Y_2Ru_2O_{7-\delta}$	0.1 M $HClO_4$	8	η_{10} = 190 mV	7
Ru@IrO_x	0.05 M H_2SO_4	2	η_{10} = 282 mV	8
3C-$SrIrO_3$ (or IrO_x/$SrIrO_3$)	0.5 M H_2SO_4	20	η_{10} = 270-290 mV	9
6H-$SrIrO_3$	0.5 M H_2SO_4	30	η_{10} = 248 mV	10
La_2LiIrO_6	0.5 M H_2SO_4		η_{10} = 350 mV	11
$Ru_{0.5}Ir_{0.5}$ oxide	0.1 M $HClO_4$		η_5 = 320 mV	12
$Sr_{0.90}Na_{0.10}RuO_3$	0.1 M $HClO_4$	~80 cycles	η_{10} = 170 mV	13
CB[6]-Ir_2	0.5 M H_2SO_4	20	η_{10} = 270 mV	14
$Rh_{22}Ir_{78}$/VX	0.5 M H_2SO_4	8	η_{10} = 292 mV	15
np-$Ir_{70}Ni_{15}Co_{15}$	0.1 M $HClO_4$	24	η_{10} = 220 mV	16
RuO_2	0.5 M H_2SO_4	1000 cycles	η_1 = 230 mV	17
$W_{0.2}Er_{0.1}Ru_{0.7}O_{2-\delta}$	0.5 M H_2SO_4	500	η_{10} = 168 mV	This work

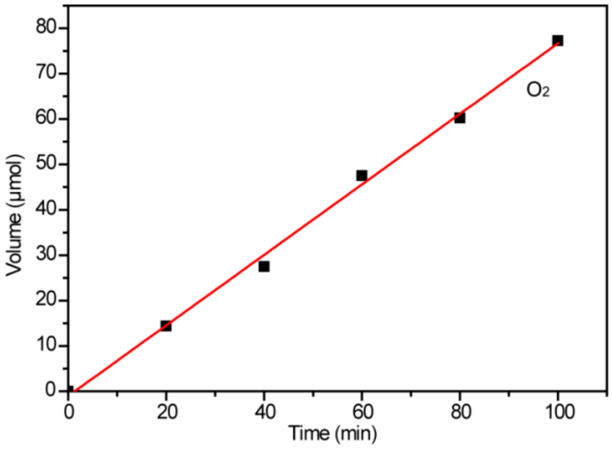

Supplementary Figure 28. **Faraday efficiency test**. The volume of O_2 generated by $W_{0.2}Er_{0.1}Ru_{0.7}O_{2-\delta}$ toward acidic OER. The red represents the fitting line, the square points represents the volume of O_2 every 20 min.

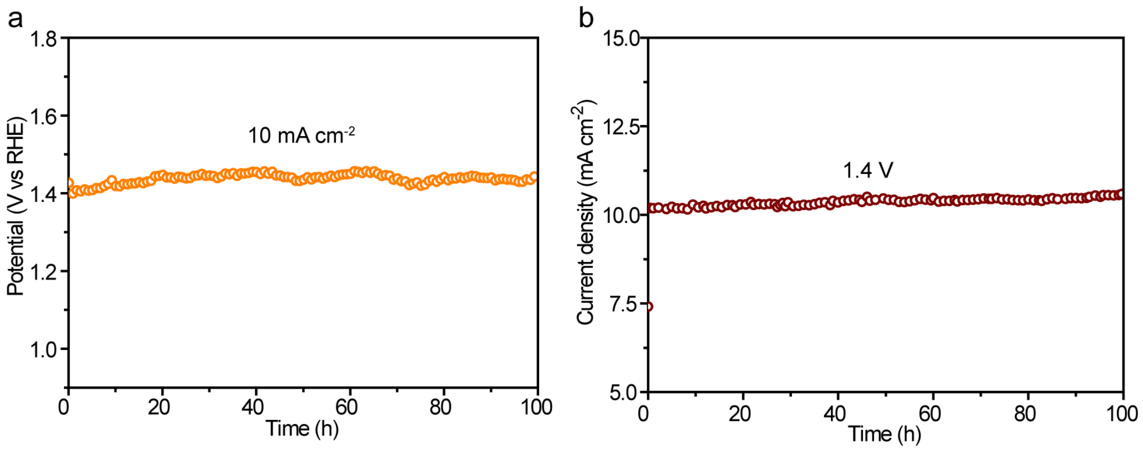

Supplementary Figure 29. **The stability of $W_{0.2}Er_{0.1}Ru_{0.7}O_{2-\delta}$ on GC**. (a) The stability of $W_{0.2}Er_{0.1}Ru_{0.7}O_{2-\delta}$ on GC at 10 mA cm^{-2}. (b) The stability of $W_{0.2}Er_{0.1}Ru_{0.7}O_{2-\delta}$ on GC at 1.4 V.

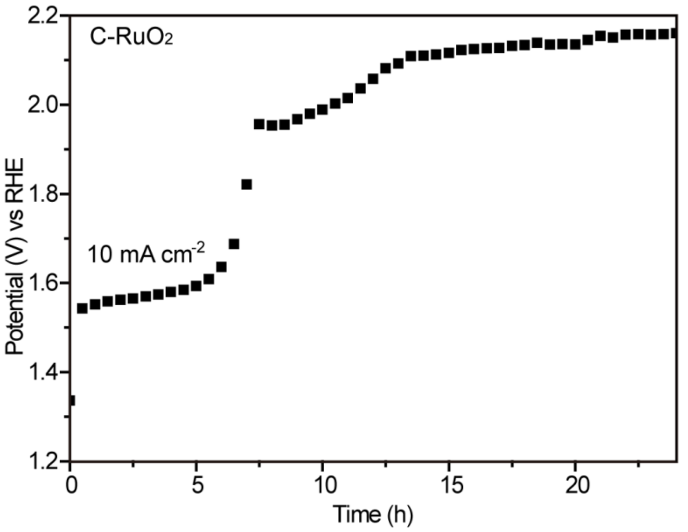

Supplementary Figure 30. **Stability of C-RuO_2**. The stability of C-RuO_2 at 10 mA·cm^{-2} in 0.5 M H_2SO_4.

ICP analysis of the concentration of each element in solution after OER.

Supplementary Table 10. ICP analysis for $W_{0.2}Er_{0.1}Ru_{0.7}O_{2-\delta}$ in the process of OER stability testing in 0.5 M H_2SO_4 at 10 $mA\cdot cm^{-2}$.

Element	After 100 h	After 300 h	After 500 h
Ru	6.0 ppb	8 ppb	11 ppb
W	1.1 ppb	1.5 ppb	1.9 ppb
Er	0.4 ppb	0.6 ppb	1.1 ppb

Supplementary Table 11. The mass of the Ru, W, and Er in $W_{0.2}Er_{0.1}Ru_{0.7}O_{2-\delta}$ after OER stability testing in 0.5 M H_2SO_4 at 10 $mA\cdot cm^{-2}$.

Element	After 100 h	After 300 h	After 500 h
Ru	0.24 μg	0.32 μg	0.44 μg
W	0.04 μg	0.06 μg	0.08 μg
Er	0.02 μg	0.03 μg	0.04 μg

Supplementary Table 12. The percentage of the dissolved Ru, W, and Er in $W_{0.2}Er_{0.1}Ru_{0.7}O_{2-\delta}$ after OER stability compared with $W_{0.2}Er_{0.1}Ru_{0.7}O_{2-\delta}$ before OER.

Element	Remaining percentage after 100 h	Remaining percentage after 300 h	Remaining percentage after 500 h
Ru	99.86%	99.8%	99.7%
W	99.97%	99.96%	99.95%
Er	99.98%	99.97%	99.96%

Supplementary Table 13. The mass of the dissolved Ru in $RuO_{2-\delta}$ after OER stability testing in 0.5 M H_2SO_4 at 10 $mA\cdot cm^{-2}$.

Element	After 50 h	After 100 h
Ru	0.2 μg	0.38 μg

Supplementary Table 14. The mass of the dissolved Ru and W in $W_{0.1}Ru_{0.9}O_{2-\delta}$ after OER stability testing in 0.5 M H_2SO_4 at 10 mA·cm^{-2}.

Element	After 50 h	After 100 h
Ru	0.11 μg	0.22 μg
W	0.03 μg	0.05 μg

Supplementary Table 15. The mass of the dissolved Ru and W in $W_{0.2}Ru_{0.8}O_{2-\delta}$ after OER stability testing in 0.5 M H_2SO_4 at 10 mA·cm^{-2}.

Element	After 50 h	After 100 h
Ru	0.13 μg	0.21 μg
W	0.03 μg	0.05 μg

Supplementary Table 16. The mass of the dissolved Ru and W in $W_{0.3}Ru_{0.7}O_{2-\delta}$ after OER stability testing in 0.5 M H_2SO_4 at 10 mA·cm^{-2}.

Element	After 50 h	After 100 h
Ru	0.14 μg	0.26 μg
W	0.05 μg	0.07 μg

Supplementary Table 17. The mass of the dissolved Ru and Er in $Er_{0.1}Ru_{0.9}O_{2-\delta}$ after OER stability testing in 0.5 M H_2SO_4 at 10 mA·cm^{-2}.

Element	After 50 h	After 100 h
Ru	0.1 μg	0.18 μg
Er	0.03 μg	0.05 μg

Supplementary Table 18. The mass of the dissolved Ru, W, and Er in $W_{0.2}Er_{0.2}Ru_{0.7}O_{2-\delta}$ after OER stability testing in 0.5 M H_2SO_4 at 10 mA·cm^{-2}.

Element	After 100 h	After 300 h	After 500 h
Ru	0.28 μg	0.68 μg	0.96 μg
W	0.05 μg	0.08 μg	0.1 μg
Er	0.03 μg	0.05 μg	0.08 μg

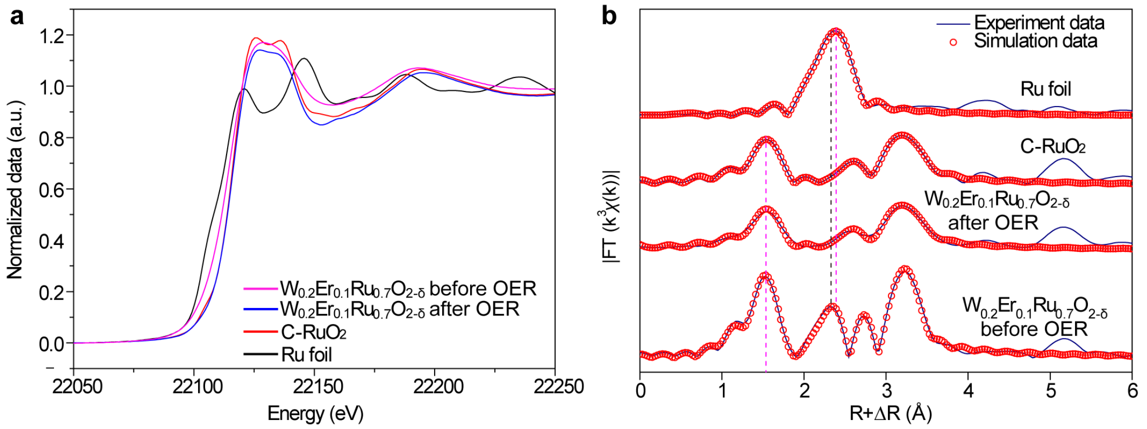

Supplementary Figure 31. **XANES and EXAFS spectra for these samples**. (a) Ru K-edge spectra for Ru foil, C-RuO_2, $W_{0.2}Er_{0.1}Ru_{0.7}O_{2-\delta}$ before and after OER, respectively. (b) FT-EXAFS spectra of Ru K-edge for Ru foil, C-RuO_2, $W_{0.2}Er_{0.1}Ru_{0.7}O_{2-\delta}$before and after OER, respectively.

Supplementary Table 19. EXAFS fitting parameters at the Ru K-edge for various samples.

Sample	Shell	N^a	R(Å)^b	σ^2(Å^2)^c	ΔE_0(eV)^d	R factor
Ru foil	Ru-Ru	12	2.68	0.0037	0.3	0.0094
RuO_2	Ru-O	6.0	1.98	0.0036	-2.5	0.0009
	Ru-Ru	1.8	3.12	0.0013		
	Ru-O	4.2	3.37	0.0036		
	Ru-Ru	3.8	3.56	0.0013		
$W_{0.2}Er_{0.1}Ru_{0.7}O_{2-\delta}$ before OER	Ru-O	5.6	1.97	0.0037	-1.9	0.0026
	Ru-Ru	1.2	2.71	0.0029		
	Ru-Ru	1.5	3.11	0.0026		
	Ru-O	2.9	3.38	0.0015		
	Ru-Ru	2.4	3.56	0.0023		
	Ru-W/Er	1.0	3.55	0.0027		
$W_{0.2}Er_{0.1}Ru_{0.7}O_{2-\delta}$ after OER	Ru-O	5.7	1.98	0.0031	-2.1	0.0022
	Ru-Ru	1.7	3.12	0.0029		
	Ru-O	3.0	3.38	0.0018		
	Ru-Ru	2.6	3.57	0.0024		
	Ru-W/Er	1.1	3.56	0.0030		

$^a N$: coordination numbers; $^b R$: bond distance; $^c \sigma^2$: Debye-Waller factors; ^{d} ΔE_0: the inner potential correction. R factor: goodness of fit. S_0^2 was set to 0.829, according to the experimental EXAFS fit of Ru foil reference by fixing CN as the known crystallographic value.

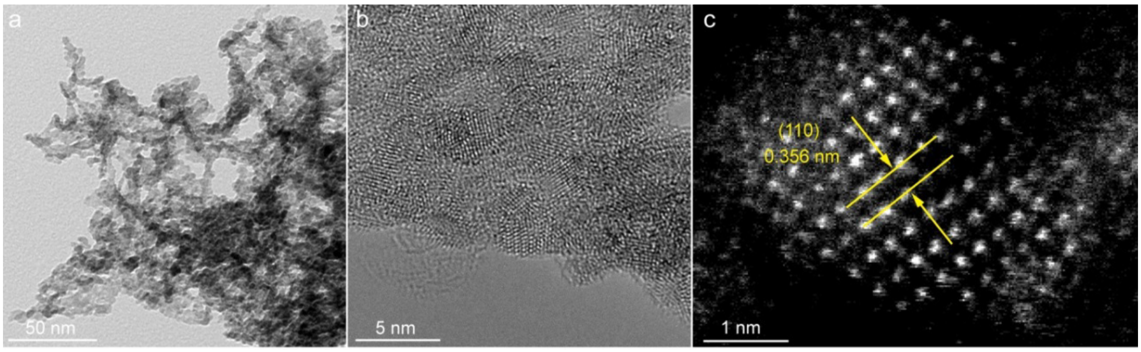

Supplementary Figure 32. **TEM characterization of $W_{0.2}Er_{0.1}Ru_{0.7}O_{2-\delta}$ after OER**. (**a**) TEM image. (**b**) HR-TEM images for the prepared $W_{0.2}Er_{0.1}Ru_{0.7}O_{2-\delta}$ nanosheets. (**c**) HR-TEM images for the prepared $W_{0.2}Er_{0.1}Ru_{0.7}O_{2-\delta}$ nanosheets.

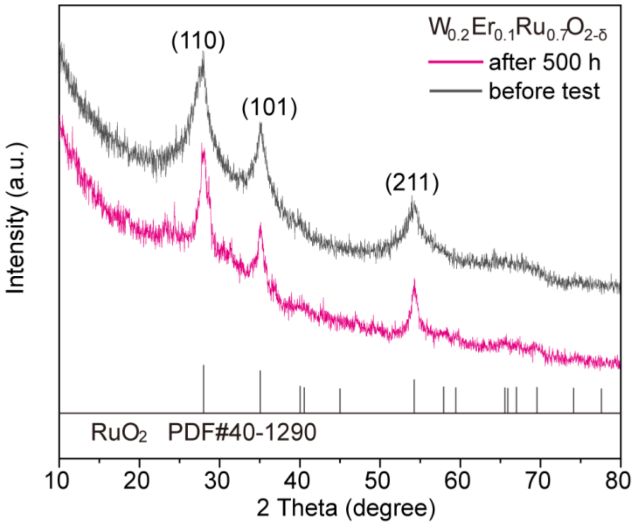

Supplementary Figure 33. **XRD pattern after OER**. XRD pattern of $W_{0.2}Er_{0.1}Ru_{0.7}O_{2-\delta}$ after OER stability was compared with that before testing.

Supplementary Note 1: The models for adsorbate evolution way toward OER

Besides the models for adsorbate evolution way toward OER, the corresponding model structures of lattice oxygen oxidation way toward OER were also established. The simulation of the two lattice oxygen atoms in $W_{0.2}Er_{0.1}Ru_1O_{2-\delta}$ participating in the reaction was established. It is expected that the blue -OH is first adsorbed on O, which was connected with Ru active site. Then, one O_2 is generated to run away and leave an oxygen vacancy. After the optimization, -OH cannot exist stably at this position and is transferred to Ru, W or Er (Supplementary Figure 2). Therefore, the lattice oxygen in $W_{0.2}Er_{0.1}Ru_1O_{2-\delta}$ will not participate in the reaction here. This result also proves that introducing W and Er would suppress the lattice oxygen participating in the reaction, in turn decreasing the dissolution rate in acidic electrolyte.

Supplementary Note 2: The compariosn for various doping locations of W in RuO_2

Theoretical calculations for different doping styles of W in RuO_2 were established and calculated. According to calculation and comparison, it can be seen that the energy barrier of PDS in $W_{0.2}Ru_{0.8}O_{2-\delta}$-1 was the smallest in these models (Supplementary Figure 5).

Supplementary Note 3: The comparison for various doping locations of Er in RuO_2

Theoretical calculations for different doping styles of Er in RuO_2 were established and calculated. The active sites in $Er_{0.1}Ru_{0.9}O_{2-\delta}$-1, $Er_{0.1}Ru_{0.9}O_{2-\delta}$-3, and $Er_{0.1}Ru_{0.9}O_{2-\delta}$-5 were on Er (Supplementary Figure 6, 7). Additionally, the active sites in $Er_{0.1}Ru_{0.9}O_{2-\delta}$-2 and $Er_{0.1}Ru_{0.9}O_{2-\delta}$-4 were on Ru. When the active sites were on Ru, the O atoms adjacent Er were greatly distorted due to the defect location. Thus, the energy barriers of PDS for $Er_{0.1}Ru_{0.9}O_{2-\delta}$-2 and $Er_{0.1}Ru_{0.9}O_{2-\delta}$-4 were much larger than that for $Er_{0.1}Ru_{0.9}O_{2-\delta}$-1, $Er_{0.1}Ru_{0.9}O_{2-\delta}$-3, and $Er_{0.1}Ru_{0.9}O_{2-\delta}$-5. According to calculation and comparison, it can be seen that the energy barrier of PDS in $Er_{0.1}Ru_{0.9}O_{2-\delta}$-1 was the smallest in these models.

Supplementary Note 4: The comparison for the subsequent layer doping in RuO_2

The models for Ru atoms replaced by Er or W in the subsequent layer were supplemented. Compared with the replaced atoms on the surface, the energy barriers for $W_{0.2}Ru_{0.8}O_{2-\delta}$-4, $Er_{0.1}Ru_{0.9}O_{2-\delta}$-6, and $W_{0.2}Er_{0.1}Ru_{0.7}O_{2-\delta}$-2 exhibited higher energy barrier of PDS than that for the established models of $W_{0.2}Ru_{0.8}O_{2-\delta}$, $Er_{0.1}Ru_{0.9}O_{2-\delta}$, and $W_{0.2}Er_{0.1}Ru_{0.7}O_{2-\delta}$ (Supplementary Figure 8).

Supplementary Note 5: The influence of neighboring intermediates around active sites

At $U > 1.23$ V vs. RHE, the surface of the prepared and established models for these RuO_2-based materials is not bare anymore. And there should be neighboring intermediates around active sites, which have influence on the energetics of the elementary processes in the OER. Thus, we tried our best to

simulate the neighboring intermediates appearing around the active sites. Firstly, –OH could be adsorbed on the active site, which was calculated in Supplementary Figure 4-8. Simultaneously, neighboring intermediates were ignored in Supplementary Figure 4-8. In the second step, one H could be taken off from the active sites. Simultaneously, the OER reaction could also be occurred on the active sites closing to the active site, which has been adsorbed –OH (Supplementary Figure 11). According to calculation, the energy barrier for PDS decreased, compared with the traditional models. Therefore, the results also indicated that the neighboring intermediates around active sites could contribute to enhancing the activity. Moreover, the free energy were calculated for the established models at U = 0, 1.23, and 1.4 V, respectively. Due to the influence of the neighboring intermediates around active sites, we only calculate limited models. In the maintext and Supplementary Figure 3-8, the calculated free-binding energies ignored the influence of the neighboring intermediates on the energetics.

Supplementary Note 6: The LSV error bars

The error bars of the current density measurement for these prepared electrocatalysts were also calculated. Firstly, each error bar represents the error among each repeated LSV curve for each sample at 10 mA cm^{-2} after 500th cycle. It can be seen from Figure S21, the LSV curves revealed that the catalysts almost kept stable after 500th cycle (Scan rate: 5 $mV{\cdot}s^{-1}$). The mean value of the overpotentials at 10 $mA{\cdot}cm^{-2}$ for these LSV curves were calculated from the 1st, 100th, 300th, and 500th circle. Simultaneously, the LSV curves shown in our main-text were all obtained after stable operation. In this part, we need to emphasize that the overpotentials at 10 mA cm^{-2} from the LSV curves need to be *i*R corrected. The value of R for correction was the same value. Sometimes, according to strict procedures, after testing each LSV curve, it is necessary to test its impedance value.

The calculated method for the error bars was illustrated as follows:

Firstly, the overpotentials of 100th, 300th, and 500th LSV curves minus the overpotential of the 1st LSV curve at 10 $mA{\cdot}cm^{-2}$. Then, the average of the difference was considered as the error (Supplementary Figure 22).

Supplementary Note 6: ECSA for bare carbon paper

ECSA for bare carbon paper (CP) was estimated by CV curves (Supplementary Figure 23). According to calculation, the ECSA for bare CP occupies 0.036% of the ECSA for the prepared $RuO_{2-\delta}$@CP (Supplementary Figure 24). Simultaneously, the ECSA for the prepared $RuO_{2-\delta}$ is the smallest one among these prepared electrocatalysts. Therefore, the influence of the carbon paper on enhancing the ECSA for

these prepared electrodes could be ignored.

Supplementary Note 7: ECSA for electrocatalysts on GC

The CV curves and ECSA for these prepared electrocatalysts loading on glassy carbon (GC) electrodes were also tested (Supplementary Figure 26, 27). The detailed data of C_{dl} for these prepared electrodes was shown in Supplementary Figure 27. According to Supplementary Figure 27, it can be seen that the C_{dl} of electrocatalysts on GC was almost consistent with that the electrocatalysts on carbon paper (Supplementary Figure 21 and Figure 6c).

Supplementary Note 8: Stability of electrocatalysts on GC

The stability of the prepared $W_{0.2}Er_{0.1}Ru_{0.7}O_{2-\delta}$ on glassy carbon (GC) electrode has been tested and added into supplementary information. As shown in Supplementary Figure 29, $W_{0.2}Er_{0.1}Ru_{0.7}O_{2-\delta}$ on glassy carbon (GC) electrode could run stably at least 100 h with a rising overpotential of 39 mV. Besides the chronopotentometric at 10 mA cm^{-2}, the stability of the prepared $W_{0.2}Er_{0.1}Ru_{0.7}O_{2-\delta}$ on GC was also tested using chronopotentometric at 1.4 V vs RHE. As shown in Supplementary Figure 29, the stability of $W_{0.2}Er_{0.1}Ru_{0.7}O_{2-\delta}$ using chronopotentometric at 1.4 V vs RHE was also as good as the test at 10 mA cm^{-2}.

Simultaneously, it could be seen that the stability of $W_{0.2}Er_{0.1}Ru_{0.7}O_{2-\delta}$ on GC electrode was very close to the stability of $W_{0.2}Er_{0.1}Ru_{0.7}O_{2-\delta}$ on carbon paper in the main-text.

Supplementary References

1. Shan J., Ling T., Davey K., Zheng Y., Qiao S.Z. Transition-Metal-Doped RuIr Bifunctional Nanocrystals for Overall Water Splitting in Acidic Environments. *Adv. Mater.* **31**, 1900510 (2019).
2. Miao X., Zhang L., Wu L., Hu Z., Shi L., Zhou S. Quadruple perovskite ruthenate as a highly efficient catalyst for acidic water oxidation. *Nat. Commun.* **10**, 3809 (2019).
3. Lin Y., *et al.* Chromium-ruthenium oxide solid solution electrocatalyst for highly efficient oxygen evolution reaction in acidic media. *Nat. Commun.* **10**, 162 (2019).
4. Cao L., *et al.* Dynamic oxygen adsorption on single-atomic Ruthenium catalyst with high performance for acidic oxygen evolution reaction. *Nat. Commun.* **10**, 4849 (2019).
5. Yao Y., *et al.* Engineering the electronic structure of single atom Ru sites via compressive strain boosts acidic water oxidation electrocatalysis. *Nat. Catal.* **2**, 304-313 (2019).
6. Su J., *et al.* Assembling Ultrasmall Copper-Doped Ruthenium Oxide Nanocrystals into Hollow Porous Polyhedra: Highly Robust Electrocatalysts for Oxygen Evolution in Acidic Media. *Adv. Mater.*, e1801351 (2018).
7. Kim J., *et al.* High-Performance Pyrochlore-Type Yttrium Ruthenate Electrocatalyst for Oxygen Evolution Reaction in Acidic Media. *J. Am. Chem. Soc.* **139**, 12076-12083 (2017).
8. Shan J., *et al.* Charge-Redistribution-Enhanced Nanocrystalline Ru@IrO_x Electrocatalysts for Oxygen Evolution in Acidic Media. *Chem* **5**, 445-459 (2019).
9. A highly active and stable $IrO_x/SrIrO_3$ catalyst for the oxygen evolution reaction. *Science* **353**, 1011 (2016).
10. Yang L., *et al.* Efficient oxygen evolution electrocatalysis in acid by a perovskite with face-sharing IrO_6 octahedral dimers. *Nat. Commun.* **9**, 5236 (2018).
11. Grimaud A., *et al.* Activation of surface oxygen sites on an iridium-based model catalyst for the oxygen evolution reaction. *Nat. Energy* **2**, 16189 (2016).
12. Using Surface Segregation To Design Stable Ru-Ir Oxides for the Oxygen Evolution Reaction in Acidic Environments. *Angew. Chem.* **126**, 14240-14245 (2014).
13. Retuerto M., *et al.* Na-doped ruthenium perovskite electrocatalysts with improved oxygen evolution activity and durability in acidic media. *Nat. Commun.* **10**, 2041 (2019).
14. You H., *et al.* Highly Active and Stable Water Splitting in Acidic Media Using a Bifunctional

Iridium/Cucurbit[6]uril Catalyst. *ACS Energy Lett.* **4**, 1301-1307 (2019).

15. Guo H., *et al.* Rational Design of Rhodium–Iridium Alloy Nanoparticles as Highly Active Catalysts for Acidic Oxygen Evolution. *ACS Nano* **13**, 13225-13234 (2019).
16. Zhao Y., *et al.* 3D nanoporous iridium-based alloy microwires for efficient oxygen evolution in acidic media. *Nano Energy* **59**, 146-153 (2019).
17. Audichon T., Napporn T.W., Canaff C., Morais C., Comminges C., Kokoh K.B. IrO_2 Coated on RuO_2 as Efficient and Stable Electroactive Nanocatalysts for Electrochemical Water Splitting. *J. Phys. Chem. C* **120**, 2562-2573 (2016).
